# *Oscarella lobularis* (Homoscleromorpha, Porifera) Regeneration: Epithelial Morphogenesis and Metaplasia

**DOI:** 10.1371/journal.pone.0134566

**Published:** 2015-08-13

**Authors:** Alexander V. Ereskovsky, Ilya E. Borisenko, Pascal Lapébie, Eve Gazave, Daria B. Tokina, Carole Borchiellini

**Affiliations:** 1 Institut Méditerranéen de Biodiversité et d’Ecologie Marine et Continentale (IMBE), CNRS, IRD, Aix Marseille Université, Avignon Université, Station Marine d’Endoume, Marseille, France; 2 Department of Embryology, Faculty of Biology, St. Petersburg State University, St. Petersburg, Russia; 3 Laboratoire de Biologie du Développement de Villefranche sur mer UMR7009 CNRS/UPMC Observatoire Océanologique Quai de la Darse, Villefranche-sur-Mer, France; 4 Institut Jacques Monod, CNRS, UMR 7592, Univ Paris Diderot, Sorbonne Paris Cité, Paris, France; National Cancer Institute, UNITED STATES

## Abstract

Sponges are known to possess remarkable reconstitutive and regenerative abilities ranging from common wounding or body part regeneration to more impressive re-building of a functional body from dissociated cells. Among the four sponge classes, Homoscleromorpha is notably the only sponge group to possess morphologically distinct basement membrane and specialized cell-junctions, and is therefore considered to possess true epithelia. The consequence of this peculiar organization is the predominance of epithelial morphogenesis during ontogenesis of these sponges. In this work we reveal the underlying cellular mechanisms used during morphogenesis accompanying ectosome regeneration in the homoscleromorph sponge model: *Oscarella lobularis*. We identified three main sources of novel exopinacoderm during the processes of its regeneration and the restoration of functional peripheral parts of the aquiferous system in *O*. *lobularis*: (1) intact exopinacoderm surrounding the wound surface, (2) the endopinacoderm from peripheral exhalant and inhalant canals, and (3) the intact choanoderm found on the wound surface. The basic morphogenetic processes during regeneration are the spreading and fusion of epithelial sheets that merge into one continuous epithelium. Transdifferentiation of choanocytes into exopinacocytes is also present. Epithelial-mesenchymal transition is absent during regeneration. Moreover, we cannot reveal any other morphologically distinct pluripotent cells. In *Oscarella*, neither blastema formation nor local dedifferentiation and proliferation have been detected, which is probably due to the high morphogenetic plasticity of the tissue. Regeneration in *O*. *lobularis* goes through cell transdifferentiation and through the processes, when lost body parts are replaced by the remodeling of the remaining tissue. Morphogenesis during ectosome regeneration in *O*. *lobularis* is correlated with its true epithelial organization. Knowledge of the morphological basis of morphogenesis during *Oscarella* regeneration could have important implications for our understanding of the diversity and evolution of regeneration mechanisms in metazoans, and is a strong basis for future investigations with molecular-biological approaches.

## Introduction

The ability to regenerate is widespread throughout the animal kingdom. It has been observed in most animal phyla but with a great variability of regenerative potentialities: from renewing single cell types such as in the case of the salamander lens [1] to complete body parts, such as in the case of planarian regeneration [2], with all possible intermediates (tissues, organs) [3,4]. The recent advances in identifying the cell sources for regeneration in several metazoan model organisms have revealed a diversity of ways in which injured tissues provide progenitor cells, demonstrating the importance of studying regeneration in many different animal and tissue contexts [5]. Based on the cellular mechanisms involved, regeneration traditionally divided into three broad categories: 1) epimorphosis, 2) morphallaxis, and 3) transdifferentiation or metaplasia [6–9]. Epimorphosis requires active cellular proliferation prior to the replacement of the lost body part. In some animals, such as cnidarians, planarians and annelids, pre-existing stem cells begin to proliferate and migrate to the damaged area in response to injury [9]. These cells then form a mass of proliferating cells, known as the regeneration blastema, which will later differentiate into the specialized cells that comprise the regenerated structure [2]. In contrast to epimorphosis, morphallaxis refers to the type of regeneration in which lost body parts are replaced by the remodeling of the remaining tissue. In this process, little or no cellular proliferation takes place during the regeneration (a well-described example of this process is *Hydra* [10]). The notable feature of morphallaxis is that the majority of regenerated tissue comes from tissue which is already present in the organism.

The third mechanism, metaplasia or transdifferentiation, is involved in regenerate formation. Transdifferentiation was defined by Okada [7] as an irreversible switch of one type of already differentiated cell to another. Transdifferentiation can happen without cell division, or via a progenitor cell produced by dedifferentiation [5,8,11]. Although transdifferentiation was mainly investigated in the context of regeneration (reviewed in [7,11,12], it can also be part of normal development in animals [12–15].

Differentiation from stem cells appears to be the key process ensuring cell renewal in all animals, while dedifferentiation and transdifferentiation are rarer [8].

To understand the evolutionary history of the diverse regeneration processes and to determine their common basis, it is necessary to study the regeneration principles of a large range of metazoan lineages. For this purpose, sponges are a suitable model. Indeed, according to recent phylogenomic analyses, sponges (Porifera) are one of the sister group of Eumetazoa (review in [16]). They are presently considered to be composed of four classes: the Hexactinellida, Demospongiae, Calcarea, and Homoscleromorpha [17]. In sponges, the tissues are simpler, both structurally and functionally, than in the other Metazoa. In particular, sponge tissues are always more multifunctional than their counterparts in advanced animals. In addition, the cells of sponge tissues possess a very high capacity for transdifferentiation into cells of other types [3,18]. Moreover, sponges possess very high reconstitutive and regenerative abilities up to the re-building of a functional body from dissociated cells (reviewed in [3,19–21]). According to histological regenerative studies, archaeocytes—amoeboid-like cells of the mesohyl—were considered to be the totipotent cells at the basis of sponge regenerative capacities. The pluripotency of archaeocytes is well-illustrated by the fact that they are not only involved in regenerative processes but are also at the origin of gametogenesis in many species [19]. More recently, the pluripotency of archaeocytes is supported by molecular data, since it has been shown that they express *Piwi*- a gene generally considered as characteristic of stem cells [22,23]. More unexpectedly, choanocytes—a specialized flagellated collar cell type playing a major role in nutrition by filtration—also express *Piwi*. Evidence for the pluripotency of choanocytes can be found during gametogenesis in some species [24], and in both Calcarea and Demospongiae choanocytes play an important role in regenerative processes [3,20,21].

The objective of this work is to reveal the underlying cellular mechanisms used during morphogenesis accompanying ectosome regeneration in our model homoscleromorph sponge *Oscarella lobularis* [25]. The aims of this study are (1) to show the variety of epithelial morphogenesis during reparative regeneration; (2) to discover the cells involved in the regeneration; and (3) to compare morphogenesis between homoscleromorphs and eumetazoans. Indeed, even if *O*. *lobularis* is capable of budding [26] and regeneration (this article), no archaeocytes have been described in this species nor in some other Oscarellidae species [27]. In the absence of archaeocytes—considered to be sponge stem cells—one can thus wonder what types of cells and mechanisms are involved in the regenerative process. In this paper, we begin to answer this question by providing a precise histological description of ectosome regeneration in the homoscleromorph sponge *O*. *lobularis* and we discuss our observations in comparison to what is known in other sponge taxa.

## Material and Methods

### Sponge materials

**Fig 1:**
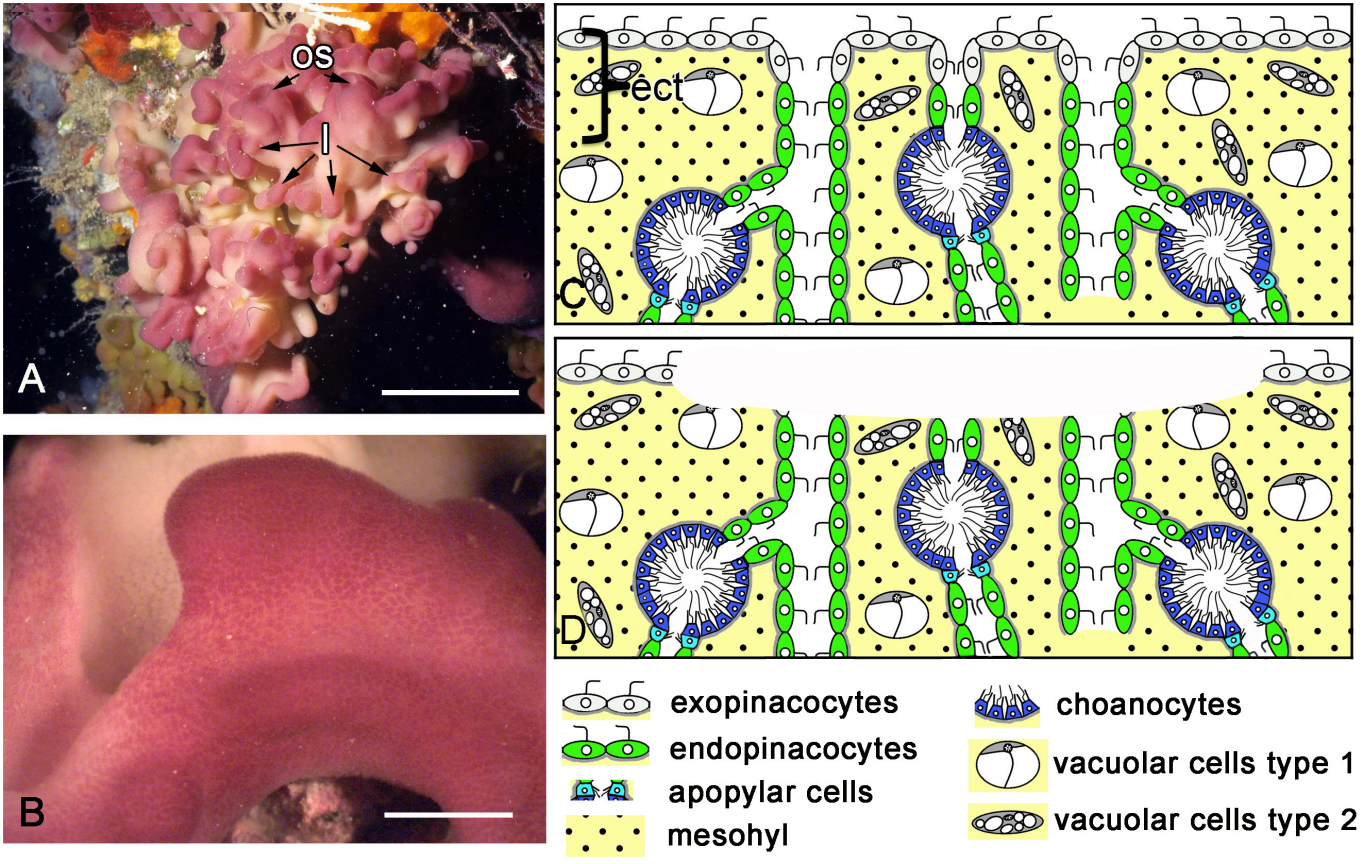
*Oscarella lobularis* habitus and simplified diagrams of its histological organization. **A**. *Oscarella lobularis in vivo* and *in situ*. **B**. Close view of a lobe before operation. Diagram of ectosome structure and cell composition before injury (**C**) and after injury (**D**). ect—ectosome. See inset for cell legends. Scale bars: A—5 cm, B—5 mm. l—lobes, os—oscula.

Specimens of *Oscarella lobularis* (Schmidt 1862), a Mediterranean species of homoscleromorph sponges (Fig 1a), were collected by SCUBA diving in June and August 2009 and 2012, in the western Mediterranean Sea, at 10–15 m depth. In the laboratory, the sponges were maintained in sea water aquaria at 16°C, in darkness.

### Surgical procedures

The apical parts of sponge lobes not bearing osculum (Fig 1b) were excised manually under a dissecting microscope using Castroviejo scissors and micro scalpels. For this reason, depth of excision differed slightly between specimens. During the amputation, not only was the ectosome (the superficial part of the sponge) excised but in addition, parts of the aquiferous system (choanocyte chambers and canals) were also removed (Fig 1c and 1d). Wounded sponges were maintained in 40 mm Petri dishes with 0.22 μm-filtered sea water. Six individuals per time were observed at various time intervals until regeneration was complete. Regeneration was monitored under a dissecting microscope and specimens were fixed 3, 6, 12, 24, 48 and 72 hours after excision.

### Light and electron microscopy

For light and electron microscopic investigation, sponge lobes were fixed for two hours in a solution composed of one volume of 2.5% glutaraldehyde, four volumes of 0.2 M cacodylate buffer and five volumes of seawater (1120 mOsm) and were post-fixed in 2% OsO_4_ in seawater at room temperature for two hours. After fixation, samples were washed in 0.2 M cacodylate buffer and dehydrated through a graded ethanol series. Specimens were embedded in Araldite resin for semithin sectioning and transmission electron microscopy (TEM). Semithin sections (1 μm in thickness) were cut on a Reichert Jung Ultramicrotome equipped with a "Micro Star" 45° diamond knife, then stained with toluidin blue, and observed under light using a WILD M20 microscope. The digital photos were made on a Leica DMLB microscope equipped with the Evolution LC color photo capture system. Ultrathin sections (60–80 nm) were cut with a Leica UCT Ultramicrotome equipped with a Drukkert 45° diamond knife. Ultrathin sections, contrasted with uranyl acetate and lead citrate, were observed under a Zeiss-1000 transmission electron microscope (TEM). For scanning electron microscopy (SEM), fixed specimens were critical-point-dried, sputter-coated with gold-palladium, and observed under a Hitachi S 570 SEM.

To calculate the dynamics of mesohylar cells (types 1 and 2 vacuolar cells) in the wound zone, we analyzed digital photographs of 10 sections for each specimen of each stage. Accordingly, four photographs (a total surveyed area of 40 mm^2^ per individual), from serial sections were taken in order to avoid any overlapping of vacuolar cells that could lead to overestimation.

### Data analysis

The calculations of mesohylar cell number (types 1 and 2 vacuolar cells) were carried out on the serial semithin sections. Six sponge specimens were used for each regeneration stage. For each specimen, we analyzed digital photos of 8 sections (four photos from four serial sections, in order to avoid overestimation due to the overlapping of cells). We determined the number of vacuolar cells throughout the regenerate stages and estimated each cell type over an area of 1mm^2^ using ImageJ Software (http://rsb.info.nih.gov/ij/index.html).

### Choanocyte lineage tracing with Indian Ink

The commercial Indian ink (Lefranc & Bourgeois) with particles diameter 50 nm was used to label choanocytes in adult sponges. Intact sponges were incubated in seawater with 2% of Indian ink during 12 h before experiments. Incubation for 12 hour was sufficient to differentially label the majority of choanocytes in sponge. Label occurred inside of phagosomes of choanocytes and newer neither in other cell types of *Oscarella lobularis* nor in the mesohyl (S1 Fig). The labelled specimens were rinsed in fresh seawater several times and then allowed to develop further. Specimens were fixed for TEM as described below at various stages of regeneration.

### Cell proliferation investigation

For each stage of regeneration, six sponges were used for DNA synthesis labeling experiments. For this purpose 500 mM stock solution of 5-Ethynyl-2'-deoxyuridine (EdU; Molecular Probes) in DMSO was added to the sea water to obtain a final concentration of 800 μM. Working concentration was established in previous experiments with another sponge [28]. Different concentrations were tried, from 10 μM according to manufacturer recommendations, to 1000 μM; the first stable signal was registered with 750 μM of EdU. This protocol of treatment was applied in trials with *Oscarella* involving two or three sponges per concentration and time point. Filtered sea water or EdU solution was replaced daily. Intact sponges were used as positive controls; they were placed simultaneously in seawater with EdU. Sponges cultivated in sea water without EdU were used as a negative control. Five sponges were used for each time point in the experiment. Time of incubation with EdU was established at 6 hours: after 3 hours of incubation labeling was very weak while after 9 hours some cells undergo divisions and closely lying pairs of labeled nuclei appears. Regenerating sponges were exposed to EdU 6 hours before end of experiment (fixation), so we used continuous labeling.

Immediately after excising, experimental and control specimens were fixed in paraformaldehyde for 1 hour, followed by washing in phosphate buffered saline (PBS). This procedure was repeated at different stages of regeneration. Fixed and rinsed specimens were blocked in 5% normal ship serum/0.05% Tween-20 in PBS at pH 7.4. Specimens were treated with Click-iT EdU Alexa Fluor 488 Imaging Kit (Molecular Probes) according to manufacturer instructions, and then incubated in mouse anti-tubulin antibody (Sigma-Aldrich) at 4°C overnight. Specimens were then rinsed three times in blocking buffer for 3–5 hours in a tube rotator and incubated in Alexa Fluor labeled anti-mouse secondary antibody (Molecular Probes). After 12 hours of incubation in the secondary antibody, specimens were rinsed and the DNA was stained with TO-PRO-3 (Molecular Probes) at concentration 1 μM in PBS for 2 hours. Rinsed pieces of the sponges were mounted in DABCO-glycerol. Images were obtained using a multiphoton confocal microscope Leica TCS SP5 MP with white laser WLL. Pictures were processed with LAS AF Lite (Leica Microsystems) and ImageJ software (http://imagej.nih.gov/ij/).

### Ethics Statement

No specific permissions were required for these locations because the study was carried outside of the national park, private land or protected area. We confirm that the field studies did not involve endangered or protected species.

## Results

### Intact sponge morphology and histology

**Fig 2:**
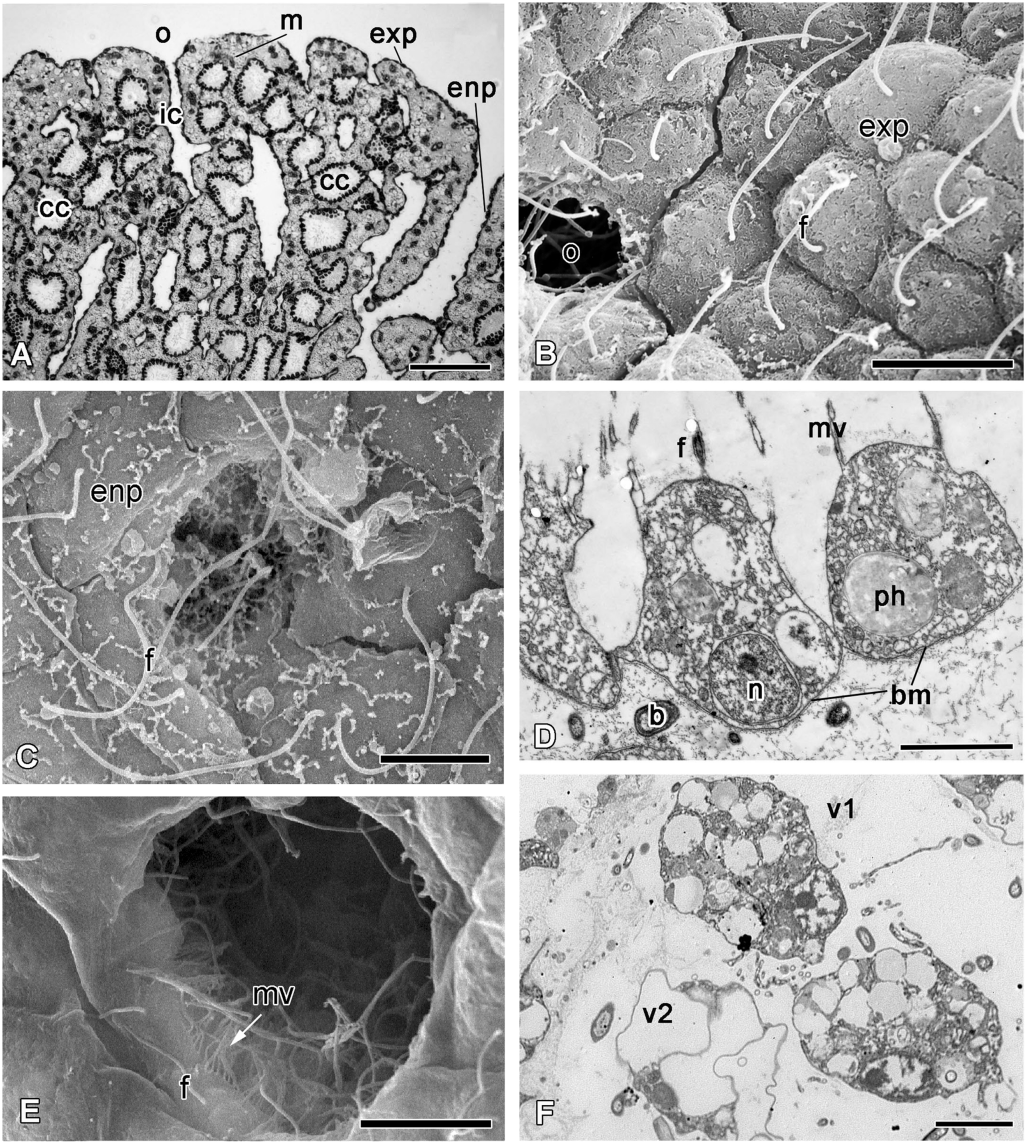
The morphology of intact *Oscarella lobularis*. **A**. Semithin section of upper part of sponge. **B**. SEM of an exopinacocytes. **C**. SEM of an endopinacocytes. **D**. TEM of a choanocytes. **E**. SEM of an apopyle with apopylar cells. **F**. TEM of type 1 and type 2 vacuolar cells. b—symbiotic bacteria, bm—basal membrane, ca—canal cavity, cc—choanocyte chamber, ec—exhalant canal, enp—endopinacocytes, exp—exopinacocytes, f—flagellum, ic—inhalant canal, m—mesohyl, mv—microvilli, n—nucleus, o—ostium, ph—phagosome, ps—pseudopodia, v—vacuole, v1—vacuolar cell type 1, v2—vacuolar cell type 2. Scale bars: A—50 μm, B—10 μm; C—5 μm; D—2 μm; E—5 μm; F—3 μm.

*Oscarella lobularis* is a soft lobulated sponge (Fig 1a) with no skeleton (neither inorganic nor organic). As is usually found in sponges, *O*. *lobularis* possesses inhalant ostia scattered evenly over the surface (Fig 2a), oscula at the top of some lobes (Fig 1a) and radial arrangement of chambers of 55–90 μm in diameter around exhalant canals (Fig 2a). A thin, unspecialized ectosome mesohyl layer (10–50 μm thickness) is situated between the surface and the aquiferous system. This sponge contains vacuolar cells of two types and symbiotic bacteria (Fig 2a and 2f). The sponge surface is lined by an even layer of flattened flagellated exopinacocytes (Fig 2b) while the aquiferous canals are lined by an even layer of flat flagellated endopinacocytes (Fig 2c). Choanocytes have a truncated shape (Fig 2d). Apopylar cells are flagellated cells with a thin apical crest of microvilli (Fig 2e) that surround the exit from the choanocyte chamber. The choanoderm and pinacoderm are lined by a basement membrane (Fig 2b and 2c), which represents an original feature in sponges. Pinacocytes are connected via specialized cell junctions that can be considered as *zonula adhaerens* [26]. Interestingly, archaeocytes are absent in the mesohyl of *O*. *lobularis*.

### Ectosome regeneration

The regeneration process can be divided into four stages:

#### Stage I. Regenerating “plug” formation (0—12h)

**Fig 3:**
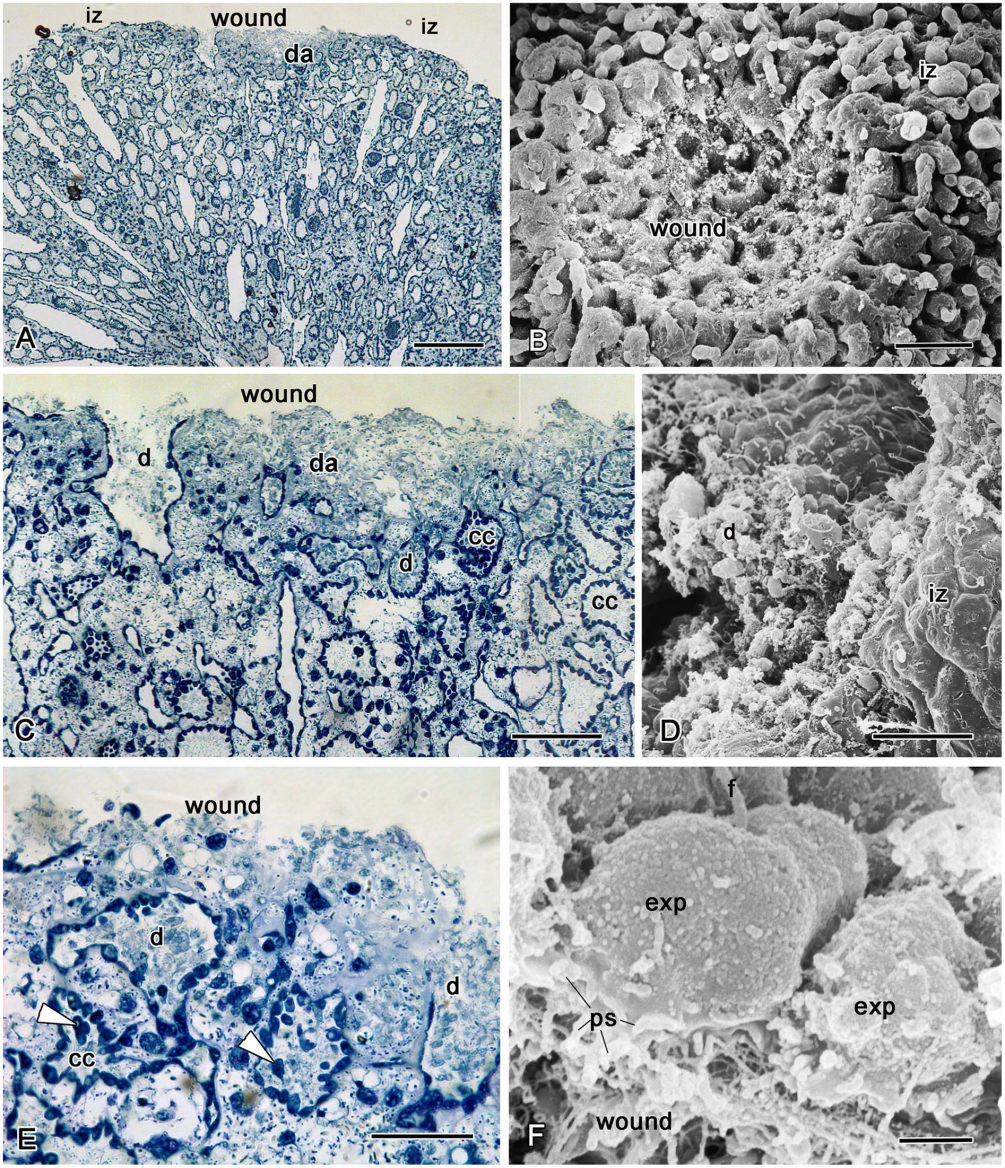
Three hours after operation. **A**. Semithin section of injured sponge. Box shows the magnified area displayed in panel **C**. **B**. SEM of the wound surface. **C**. Semithin section of a wound. **D**. SEM of the marginal zone of a wound and intact exopinacoderm. **E**. Semithin section showing the condensation of the extracellular matrix of mesohyl, collagen fibers and concentration of symbiotic bacteria in the wound, and choanocytes having migrated inside chamber cavities (arrowhead). **F**. SEM of intact exopinacocytes of marginal zones that have begun preparation for migration in the direction of the wound surfaces. This preparation is expressed in changes in the form of cells: from flat to oval and pseudopodia formation, directed toward the wound surface. cc—choanocyte chamber, d—debris; da—damaged area, exp—exopinacocyte, iz—intact zone, ps—pseudopodia. Scale bars: A— 200 μm, B—250 μm; C—100 μm; D—25 μm, E—50 μm; F—2 μm.

**Fig 4:**
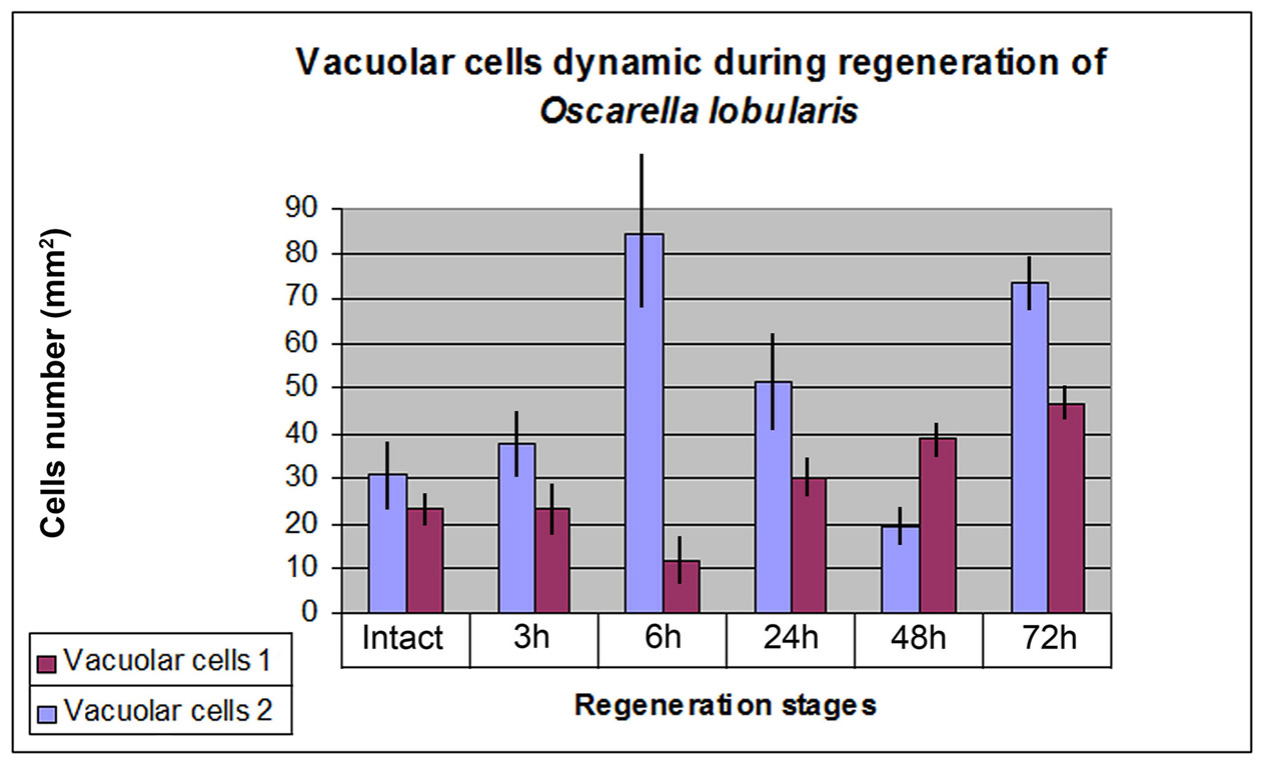
Diagram of type 1 and 2 vacuolar cell dynamics during *Oscarella lobularis* ectosome regeneration.

Immediately after excision, the area of the wound surface decreased slightly due to the mechanical contraction of the edges of the wound (Fig 3a and 3b). During the first three hours, the wound surface is full of slime and debris, consisting of microbes and fragments of cells (Fig 3c and 3d). During this initial three-hour period, the mesohyl of the ectosome in the wound area seems to be more condensed in comparison with the internal choanosome area (Fig 3a and 3c), due to condensation of the extracellular matrix, collagen fibers and a higher concentration of symbiotic bacteria (Fig 3e). The small canals of the aquiferous system in contact with the wound become isolated, and the larger ones reduce in diameter (Fig 3c and 3e). Cavities of peripheral aquiferous system canals and choanocyte chambers adjacent to a wound contain debris (Fig 3c–3e). Some of the endopinacocytes have large phagosomes. The shape of intact exopinacocytes surrounding the wound changes from flat to oval (compare Figs 3f and 2b). Type 2 vacuolar cells concentrate in the wound zone, while the average number of type 1 vacuolar cells appears unchanged (Fig 4).

The epithelial structure of the choanoderm within the choanocyte chambers is broken because of the destruction of the contacts between choanocytes. Choanocytes are enlarged, inflated and some of them migrate inside the chamber cavities (Fig 3e). The choanocytes of these chambers actively participate in phagocytosis and in the digestion of debris.

**Fig 5:**
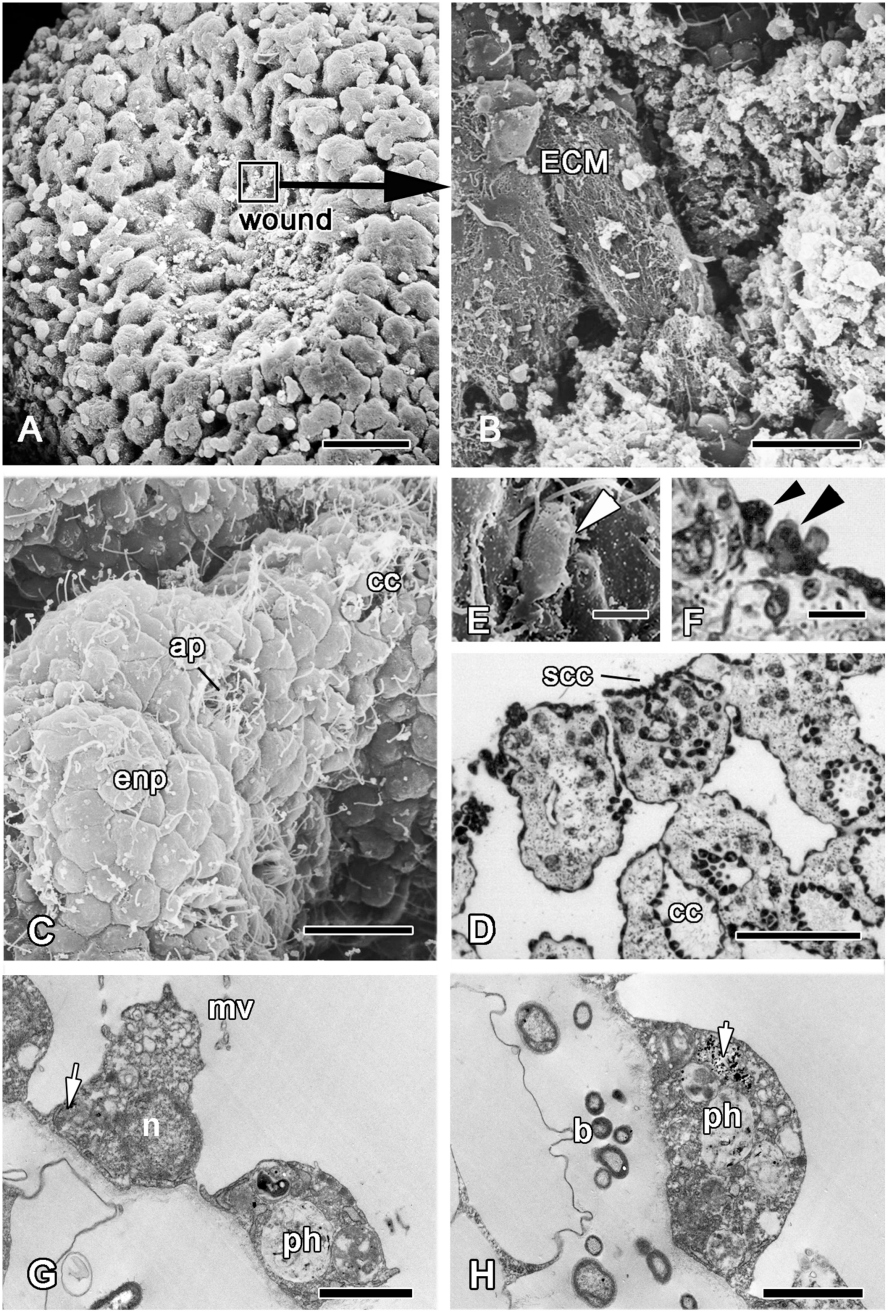
Six hours of regeneration. **A**. SEM of the wound surface. **B**. SEM of wound surface covered by ECM layer. **C**. SEM of the apopyles with apopylar cells and choanocyte chamber remaining on the wound surface. **D**. Semithin section of wound surface with spread choanocyte chamber (scc). SEM (**E**) and semithin (**F**) section of single dedifferentiated choanocytes (arrowhead), that appeared on wound surfaces. **G, H**. TEM of choanocytes labeled with Indian ink (arrows) beginning of dedifferetntiation at the wound surface. ap—apopyle, b—symbiotic bacteria, cc—choanocyte chamber, enp—endopinacocytes, ECM—extracellular matrix, mv—microvilli, ph—phagosome, n—nucleus, scc—sectioned choanocyte chamber. Scale bars: A—25 μm, B, C, D—15 μm; E, F—5 μm, G—100 μm, H—50 μm.

After six hours the greater part of the wound surface is free of debris (Fig 5a). The edges of the wound converge and its surface area decrease slightly due to the expansion of the surrounding epithelial cell sheet. Nevertheless, in the areas with damaged endopinacocytes and choanocytes, the wound surface is covered by an extra cellular matrix (ECM) in six hours. The main components of this matrix are collagen fibers (Fig 5b). The deeper areas of the wound surface are covered by endopinacocytes of peripheral canals of the aquiferous system (Fig 5c). Apopyles with apopylar cells still remain open and clearly visible on the wound surface (Fig 5c). Some damaged choanocyte chambers, which were cut off during the operation, can be seen at the surface (Fig 5d). Some choanocytes or groups of choanocytes on wound surfaces are beginning to dedifferentiate (Fig 5e–5h). They have lost contact with each other and have changed in shape from trapeziform to spherical. Their collar of microvilli is reduced or has disappeared, and the flagellum has been resorbed (Fig 5g and 5h). Dedifferentiated choanocytes as well as intact ones keep the particles of Indian ink in their phagosomes (Fig 5g and 5h).

During the three to six hour period, an active migration of type 2 vacuolar cells occurs in the wound zone. Their concentration rises more than 2.5 times (. 4). At the same time, concentration of type 1 vacuolar cells decreases twofold (4).

#### Stage II. Wound healing (12–24 h)

**Fig 6:**
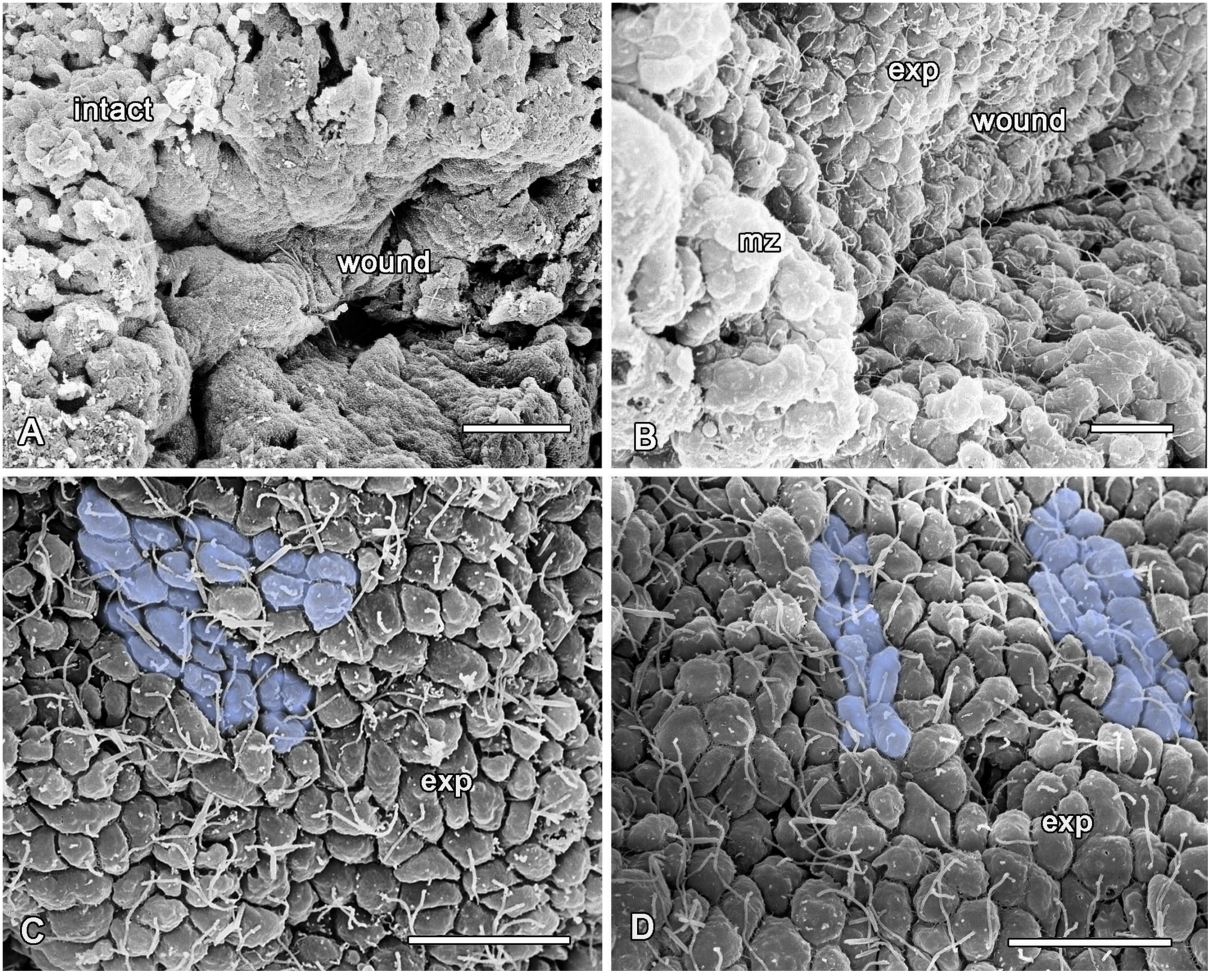
SEM images of the wound surface and new exopinacoderm after 24 hours of regeneration. **A**. A wound surface. **B**. A wound surface and marginal zone (mz). **C, D**. The new exopinacoderm with the cell "streams" (cells are artificially coloured to accentuate the "streams"). exp—exopinacocytes. Scale bars: A—D—20 μm.

**Fig 7:**
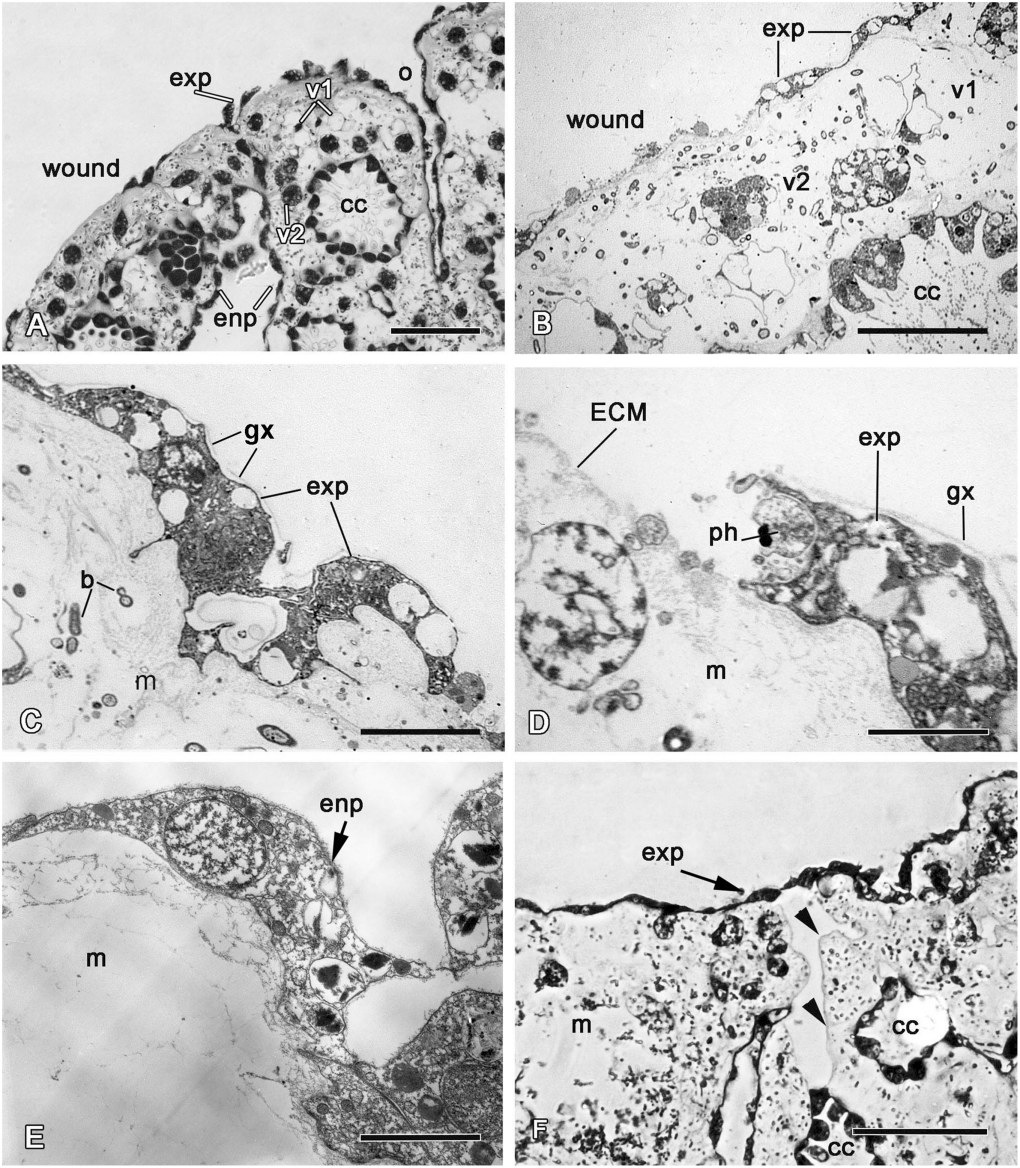
24 hours of regeneration. **A**. Semithin section of a wound surface during an epithelization by exopinacocytes, showing some small areas at the surface with no epithelium. **B**. TEM image of a wound surface covered with a dense ECM layer. **C**. TEM of the exopinacocytes of new exopinacoderm. **D**. TEM of the exopinacocyte that phagocytes a particle of cell debris at the sponge surface. **E**. TEM of the endopinacocytes of adjacent aquiferous system canals to the regenerate, which are migrating at the wound surface. **F**. Semithin section of the regenerate with new exopinacoderm and intact basement membrane (arrowhead) underlining the migrating sheet of endopinacoderm. b—symbiotic bacteria; cc—choanocyte chamber, ECM—extracellular matrix, enp—endopinacocytes, exp—exopinacocytes, gx—glycocalyx; m—mesohyl, o—ostium, ph—phagosome, v1—vacuolar cells type 1, v2—vacuolar cells type 2. Scale bars: A—50 μm, B—20 μm; C—5 μm; D, E—2 μm; F—50 μm.

At this stage, the wound surface is almost entirely covered by a layer of exopinacocytes (Fig 6). Nevertheless, there remain some small areas at the surface which lack an epithelium but which are covered with a dense ECM layer (Fig 7a and 7b).

The shapes of the exopinacocytes of the regenerative membrane are not homogenous, presenting an external surface that can be round, oval, extended—or in very rare cases, polygonal, which is typical for intact epithelium (Fig 6b). Another difference between the regenerated and the intact exopinacoderm is the absence of a relatively thick glycocalyx layer at its surface. Furthermore, the exopinacocytes of the regenerate show active movement with frequent cellular streams (or coherent cell movements) in the shape of two or three cells extended in one direction (Fig 6c and 6d). Some new exopinacocytes have oval shape, big nucleolated nucleus and abundant vacuolar inclusions.

During this period, partial crawling of the sheet of exopinacocytes from intact zones towards a regenerate occurs. This mode of epithelization is carried out by the sliding of an exopinacocyte layer through the marginal zone to regenerate over the ECM layer (Fig 7b). The moving exopinacocytes develop a leading edge with pseudopodia and filopodia, which extends towards the cells of the opposing part. The cells of both margin zones establish cell-cell contacts. During this movement, the exopinacoderm maintains both intercellular junctions and a superficial layer of the glycocalyx (Fig 7c). In the course of this convergent extension, the leading edge of the exopinacocytes can phagocyte the last remnants of cell debris (Fig 7d).

Another source of the new exopinacoderm is the endopinacocytes of adjacent aquiferous system canals. These cells migrate as an intact sheet to the regenerate surface. During this process their basal surface flattens due to a basal pseudopodia contraction, as in the case of the exopinacoderm (Fig 7e). It is noteworthy that the basement membrane underlining the migrating sheet of the endopinacoderm remains intact subsequent to cell layer migration (Fig 7f). Before this process, the adjacent choanocyte chamber is isolated from the canal by closure of its contact with the canal (Fig 7f).

**Fig 8:**
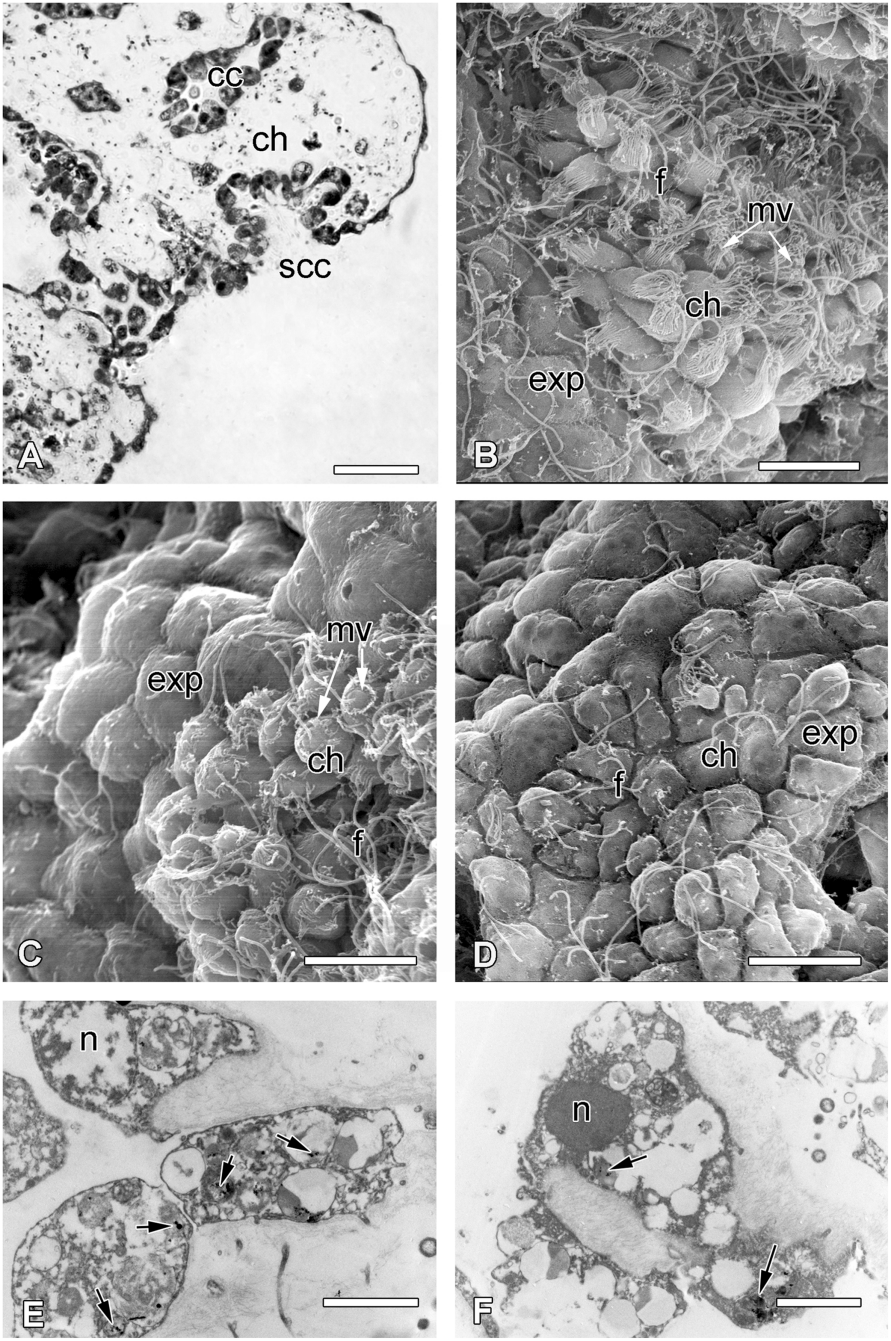
Transdifferentiation of the choanocytes and successive steps of choanocyte layer transdifferentiation into a new exopinacoderm. **A**. Semithin section of wound surface with sectioned choanocyte chamber (scc). **B**. SEM of spread choanocyte chamber showing the beginning of choanocytes transdifferentiation into exopinacocytes. **C**. SEM of spread choanocyte chamber showing intermediate stage of choanocytes transdifferentiation within the exopinacocytes. **D**. SEM of spread choanocyte chamber showing the last stage of choanocytes transdifferentiation within the exopinacocytes. **E**. TEM of transdifferentiated choanocytes after labeling in Indian ink (arrows). F. TEM of new exopinacocyte with particles of Indian ink (arrow). cc—choanocyte chambers, exp—exopinacocytes, f—flagella, n—nucleus. Scale bars: A—25 μm; B—D—10 μm; E, F—2 μm.

**Fig 9:**
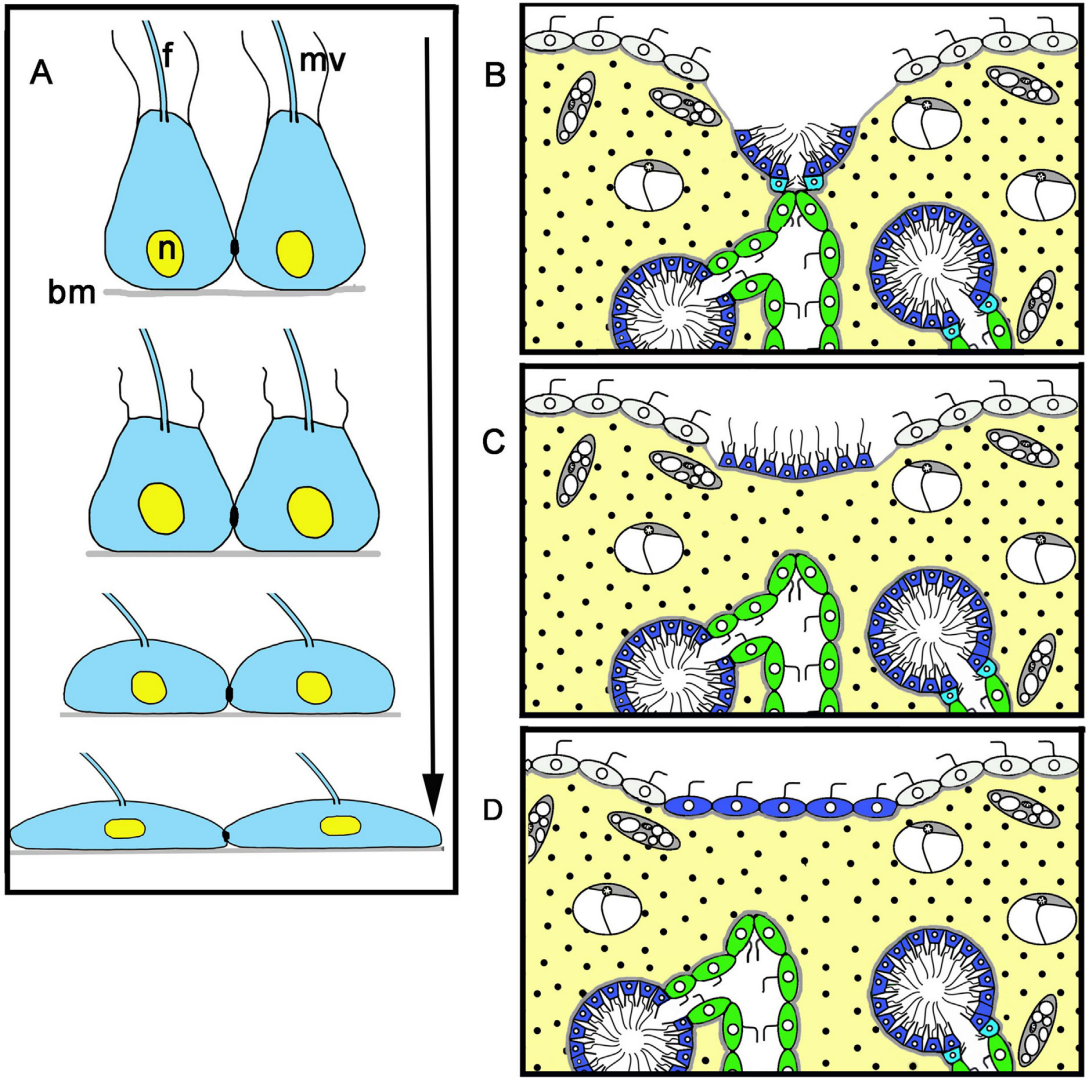
Diagram of transdifferentiation of the choanocytes (A) and successive steps of choanocyte layer transdifferentiation into a new exopinacoderm (B-D). See Fig 1 inset for the legends of (**B**). bm—basal membrane, f—flagellum, mv—microvilli, n—nucleus.

Some intact choanocyte layers appeared at a wound surface (Figs 5d and 8a) and were able to transdifferentiate directly into a new exopinacoderm. The first step of choanocyte flattening is accompanied by cell dedifferentiation. Cell shape changes from trapeziform to cubical or oval, and then to flat along the apical-basal axis, microvilli are reduced (Figs 8b–8d and 9a). The choanocyte flagellum and cell adhesion is maintained during this transdifferentiation (Fig 8b–8d). The extension of the surface area of the cell layer is the consequence of this morphogenesis (Fig 9b–9d). Some new exopinacocytes as well as transdifferentiated choanocytes at this stage have Indian ink inclusions in their phagosomes (Fig 8e and 8f). These particles present in the intact choanocytes and in the choanocytes at different stages of their transdifferentiation into exo- and endopinacocytes.

The concentration of type 2 vacuolar cells reduces noticeably while at the same time, the concentration of type 1 vacuolar cells nearly triples (Fig 4).

#### Stage III. Ectosome regeneration (24–48 h)

**Fig 10:**
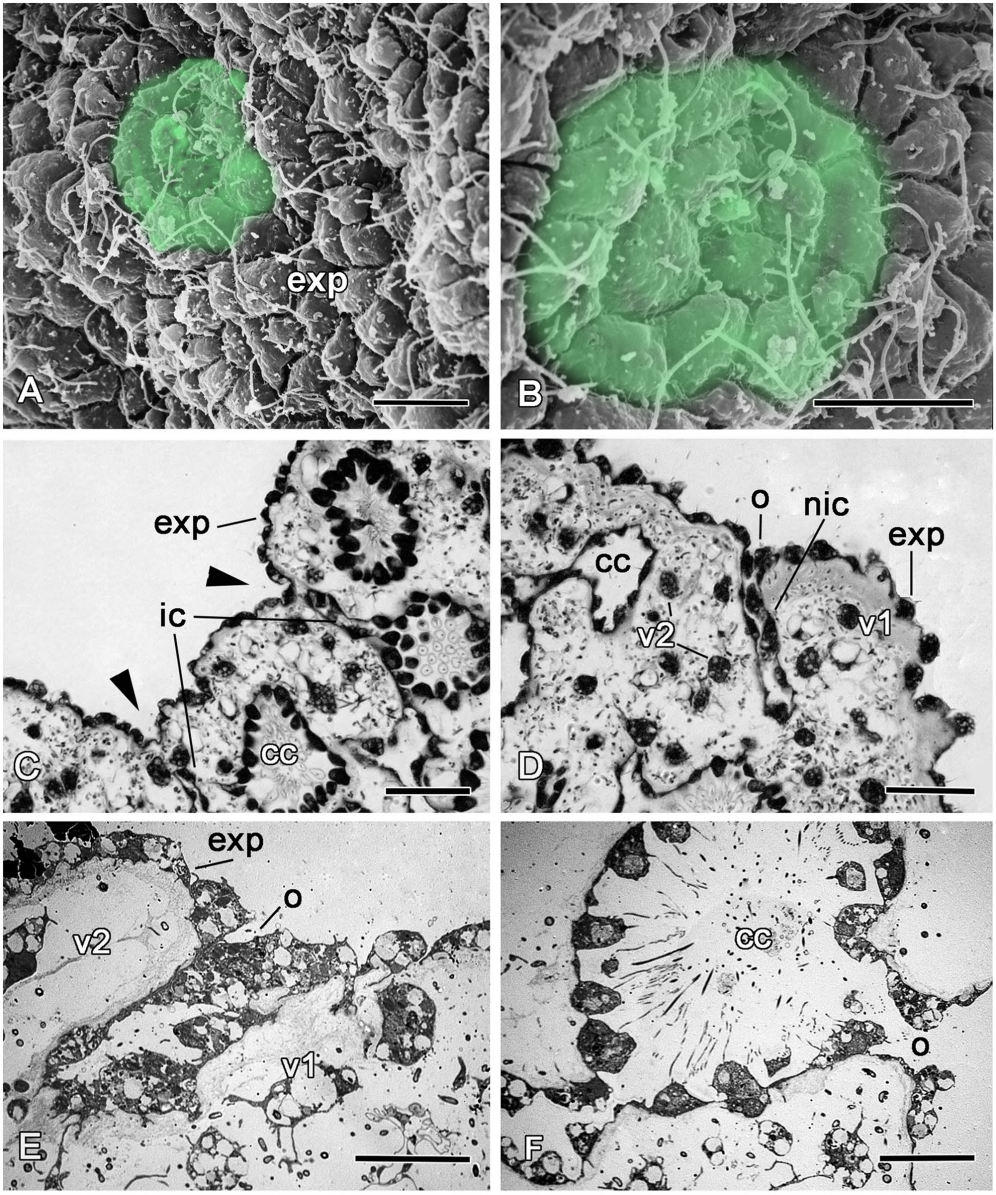
24 hours of regeneration and new ostia formation. **A, B**. SEM images of small "rosettes" of exopinacocytes on a regenerate surface (cells are artificially coloured to accentuate). **C, D**. Semithin sections of different stages of ostia formation by exopinacoderm invagination (arrowhead). **E**. TEM image of exopinacoderm invagination. **F**. TEM of new ostia connected with a choanocyte chamber. cc—choanocyte chambers, exp—exopinacocytes, ic—inhalant canal, m—mesohyl, nic—new inhalant canal, o—ostia, v1—type 1vacuolar cells, v2—type 2 vacuolar cells. Scale bars: A, B—10 μm; C, D—25 μm, E, F—10 μm.

During this third phase of regeneration the ectosome of the regenerated area begins to restore its normal structure: the proportion of mesohyl/aquiferous system reaches that found in an intact sponge (Fig 7a). Choanocyte chambers exhibit normal shape and begin to connect with the inhalant and exhalant canals (Fig 10). The major number of choanocytes keeps the particles of Indian ink in their phagosomes.

During this stage the new ostia of the regenerate start their development, when new ostia develop from small "rosettes", consisting of 5–12 exopinacocytes (Fig 10a and 10b). This formation of "rosettes" begins simultaneously in several regions of a regenerate. In the center of these "rosettes" a small pit is formed, which gradually deepens through invagination (Fig 10c–10e), before fusing with the nearest aquiferous system canals, or with adjoining choanocyte chambers (Fig 10f).

**Fig 11:**
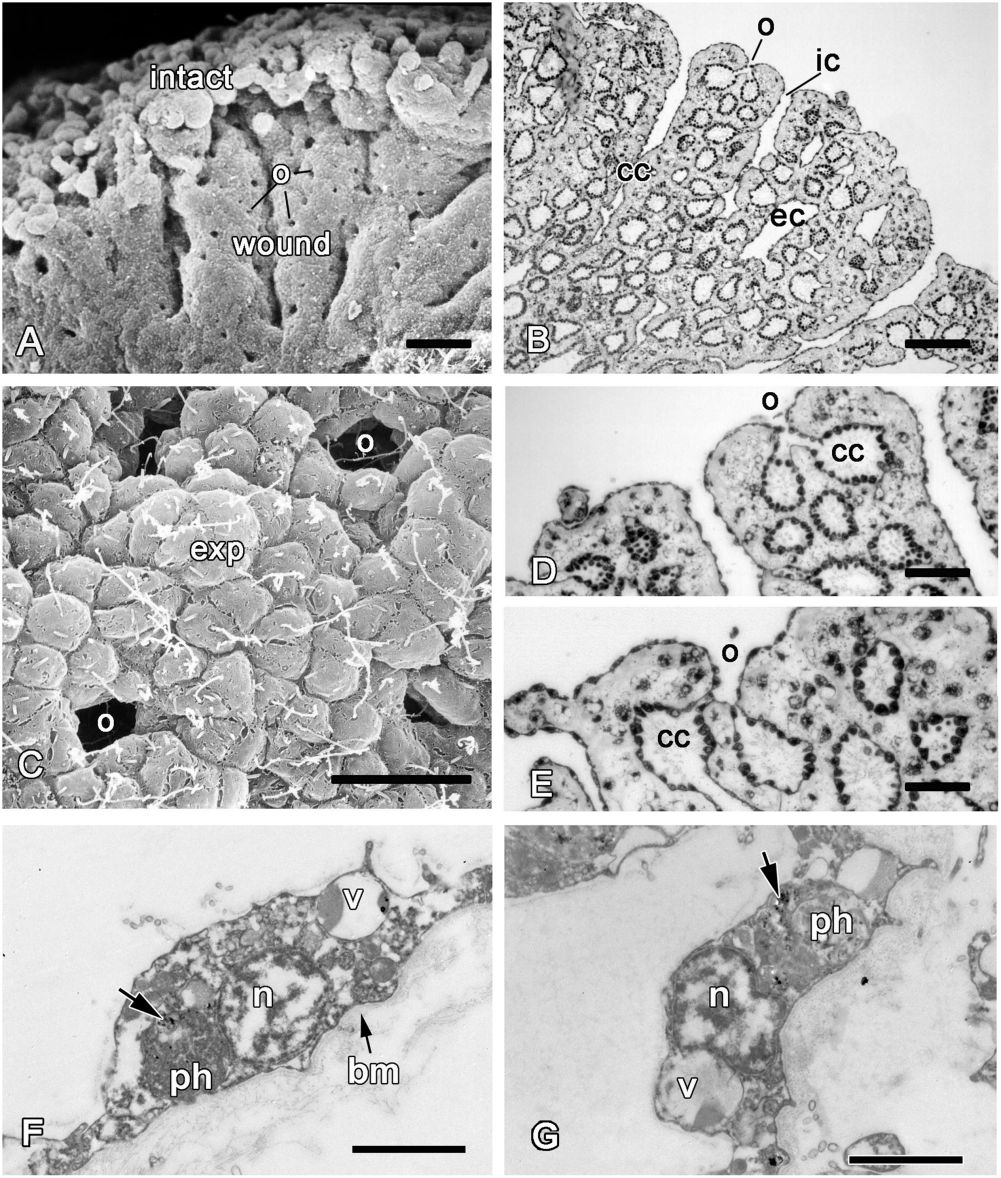
48 hours of regeneration. SEM (**A**) and semithin section (**B**) of new functioning regenerate (exopinacoderm and choanoderm). **C**. SEM image of new exopinacoderm with ostia. **D, E**. Semithin section of regenerated ectosome with functioning ostia. **F**, TEM of a new exopinacocyte with Indian ink particles (arrow) inside of phagosome. **G**. TEM of a new endopinacocyte with Indian ink particles (arrow) inside of phagosome. bm—basement membrane, cc—choanocyte chambers, ec—exhalant canal, exp—exopinacocytes, ic—inhalant canal, n—nucleus, o—ostia, ph—phagosome, v—vacuole. Scale bars: A, B—150 μm; C—20 μm; D, E—10 μm; F, G—2 μm.

After two days it can be said that the regenerating portion presents functioning exopinacoderm with ostia and inhalant canals (Fig 11). The diameter of new ostia varies from 6 to 23 μm, and they are normal in structure (Fig 11c–11e).

The surface of the regenerate is smooth, whereas the surface of intact exopinacoderm is covered by small spherical or papillary digitations of epithelium (Fig 11a). However, as is the case in intact sponges, a layer of glycocalyx covers new exopinacoderm. New exopinacoderm consists of exopinacocytes which are normal in size and polygonal in shape (Fig 11c). Some of them have Indian ink particles in the phagosomes, which they inherited from the choanocytes during their transdifferentiation. Others characterized by presence of numerous heterogeneous vacuoles (Fig 11f). The adhesive contacts between the cells are characteristic of intact sponges.

The structures of both the choanosome and ectosome, adjoining the regenerate section are in general, restored (Fig 11b). Some endopinacocytes of regenerate showed the particles of Indian ink in the phagosomes that could testify their choanocyte origin (Fig 11g). Nevertheless, the concentration of vacuolar cells of both types does not correspond to normal state (Fig 4). Indeed, the number of type 1 vacuolar cells increases, while type 2 cells actually decrease in quantity (Fig 4). The dense congestions of type 2 vacuolar cells remain in some areas only.

#### Stage IV. Choanosome regeneration (72 h)

**Fig 12:**
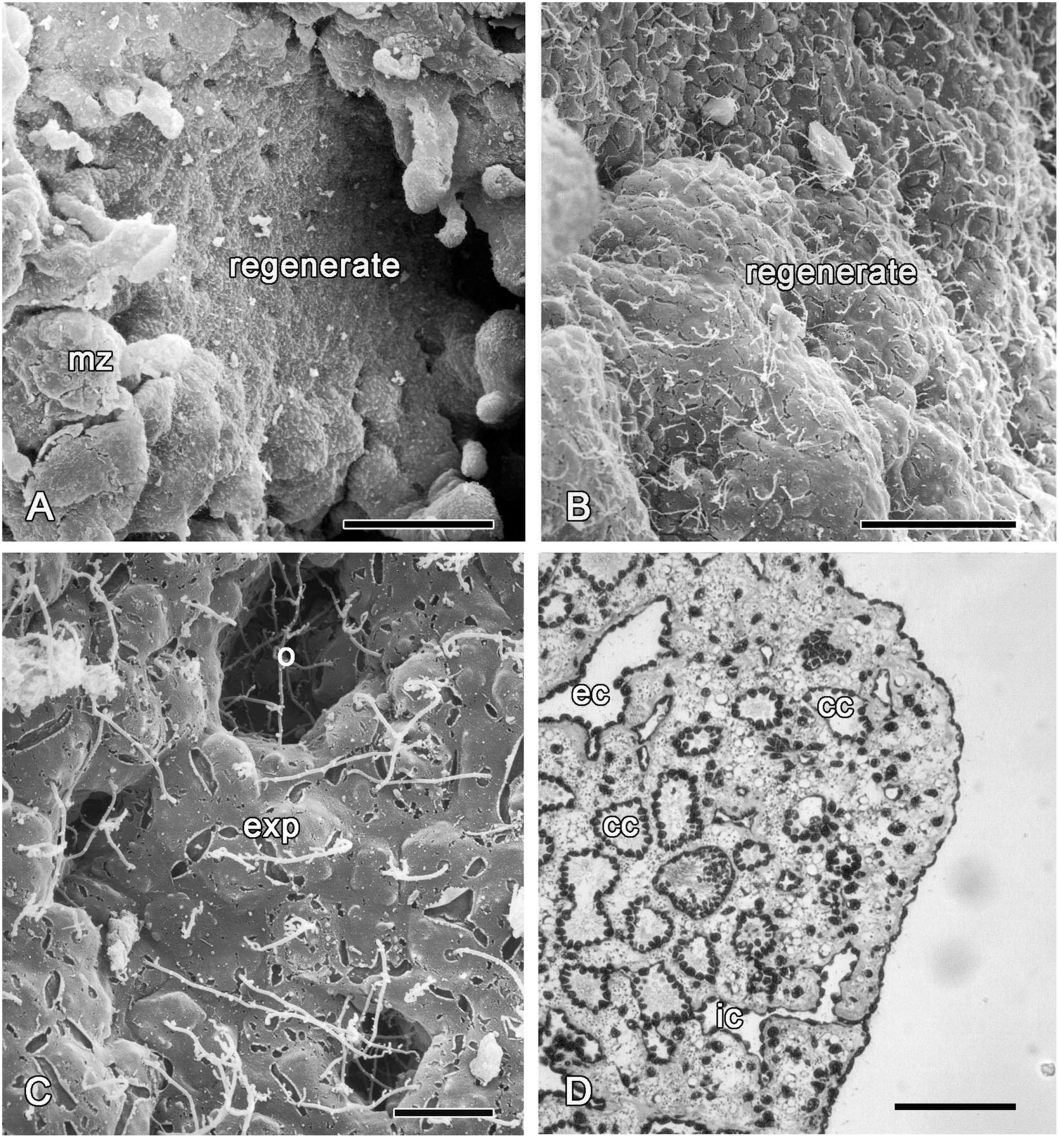
72 hours of regeneration. **A**. SEM of the regenerate and intact marginal zone. **B**. SEM of a new exopinacoderm. **C**. SEM of new exopinacoderm with the ostia and exopinacocytes, covered with a layer of glycocalyx. **D**. Semithin section of regenerated ectosome. cc—choanocyte chambers, ec—exhalant canal, exp—exopinacocytes, ic—inhalant canal, mz—intact marginal zone, o—ostia. Scale bars: A—50 μm, B—20 μm; C—10 μm; D—100 μm.

After three days, the regenerated area differs from intact zones only by its smoother surface (Fig 12a and 12b). New ostia (Fig 12c) continue to develop by local invagination of groups of exopinacocytes. The proportion of type 1 and type 2 vacuolar cells corresponds to the normal state (0.6:1), but their concentration remains high (Figs 4 and 12d).

Throughout the regeneration process, no transdifferentiation of mesohylar cells to epithelial cells (exopinacocytes, endopinacocytes, choanocytes) were observed.

### Cell proliferation dynamics

**Fig 13:**
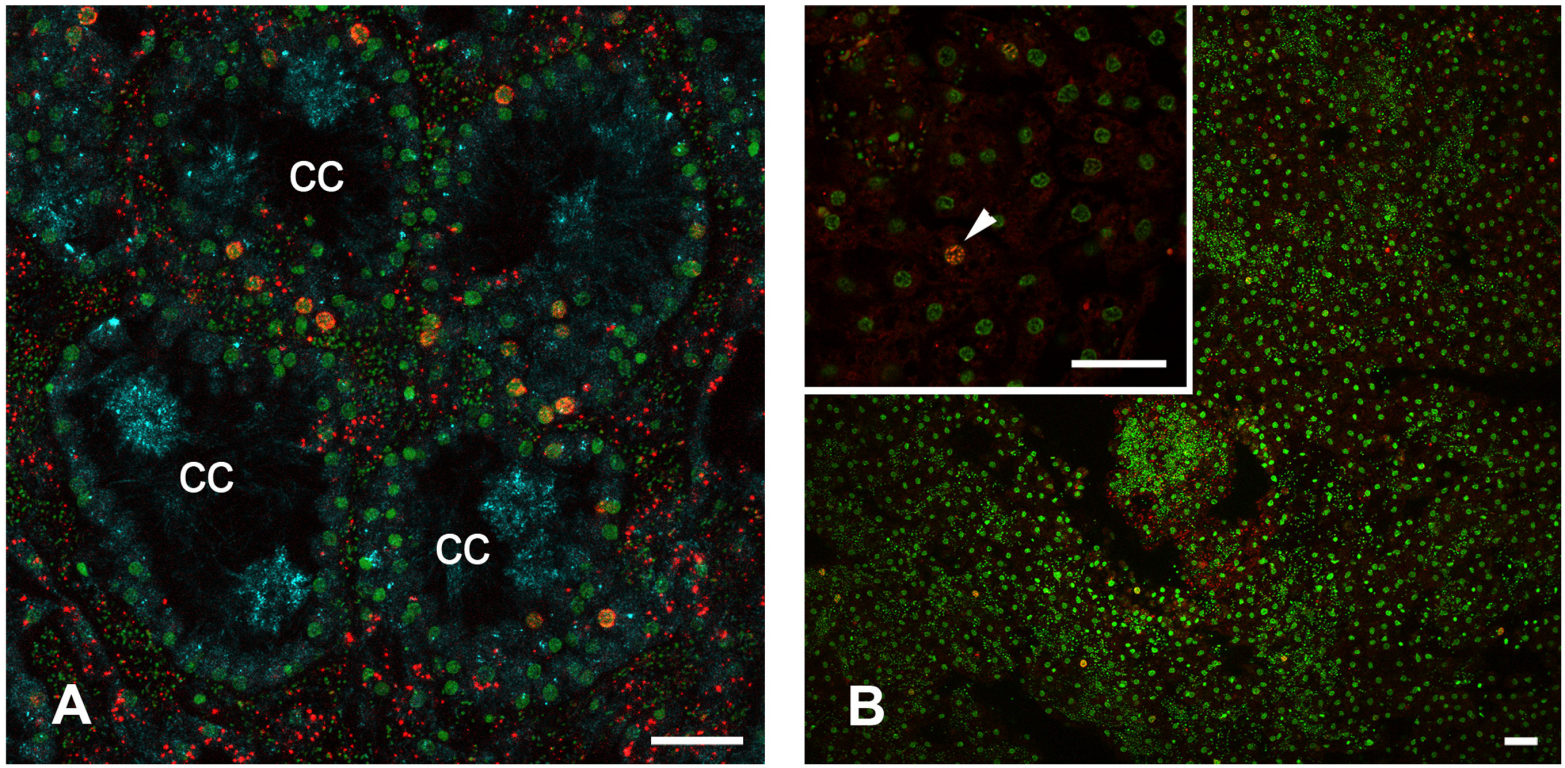
DNA synthesis in unwounded *Oscarella lobularis* (6 hours incubation with EdU); Z-stacks of confocal sections. **A**. DNA synthesis in choanocytes. **B**. DNA synthesis in exopinacocytes. Insert: part of the exopinacoderm—labeled nucleus marked with arrowheads. EdU is red, DNA is green, tubulin is blue. cc—choanocyte chamber. Scale bar—15 μm.

In unwounded sponges, EdU positive cells are present in both choanocytes (Fig 13a) and pinacocytes (Fig 13b). Choanocytes seem to be a more actively proliferating cell population. Flat exopinacocytes form random patterns of nuclei when stained with DNA-binding dye, but only one or two among these may be EdU-positive in the field of observation.

**Fig 14:**
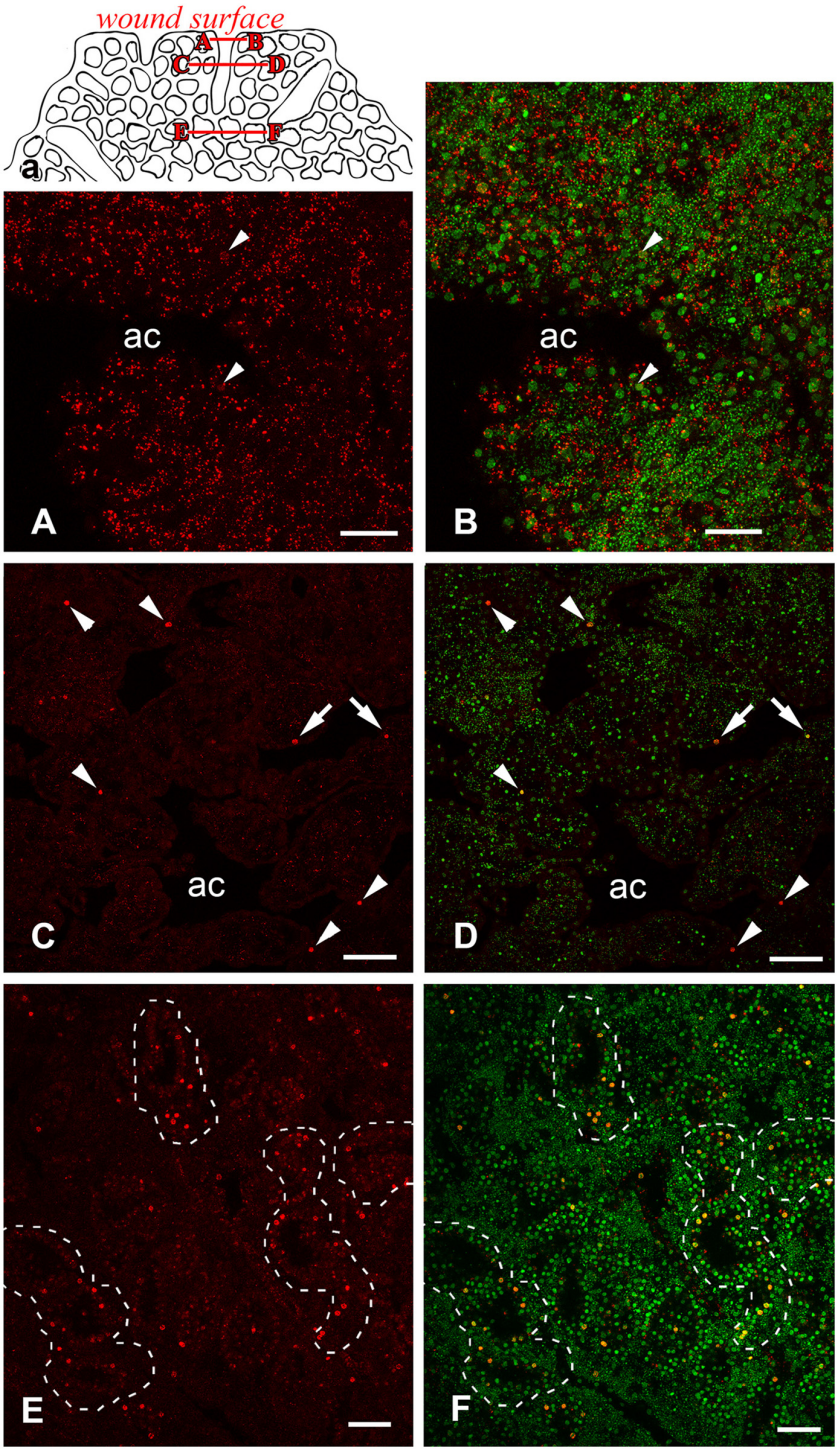
DNA synthesis in *Oscarella lobularis* during regeneration (stage I, 6 hours); confocal sections of the same specimen at different levels. Left panels—EdU, right panels—combined colors. **a**, diagram of confocal sections of sponge. **A, B**: wound surface with crossed aquiferous canals at the level A-B (depth 5 μm). **C, D**: cross section of wounded sponge at level C-D (depth 30 μm). **E, F**: deep part of sponge body at level E-F (depth 75 μm). EdU is red, DNA is green. Arrowheads indicate labeled nuclei of choanocytes, arrows indicate labeled nuclei of pinacocytes, choanocyte chambers outlined at panels E-F. ac—aquiferous canal. Scale bars: A, B—15 μm, C, D—30 μm; E, F—30 μm.

We also studied the cell proliferation profile of regenerating sponges at all stages of regeneration. Comparison of distribution patterns of EdU-positive cells in unwounded sponges and during regeneration does not show any local change of proliferation activity. For example during stage 1, after approximately six hours of regeneration, the portion of EdU labeled nuclei and the intensity of labeling does not change in wound tissue (Fig 14a–14d), although deeper choanocyte chambers that were not injured show numerous intensively labeled nuclei (Fig 14e and 14f).

**Fig 15:**
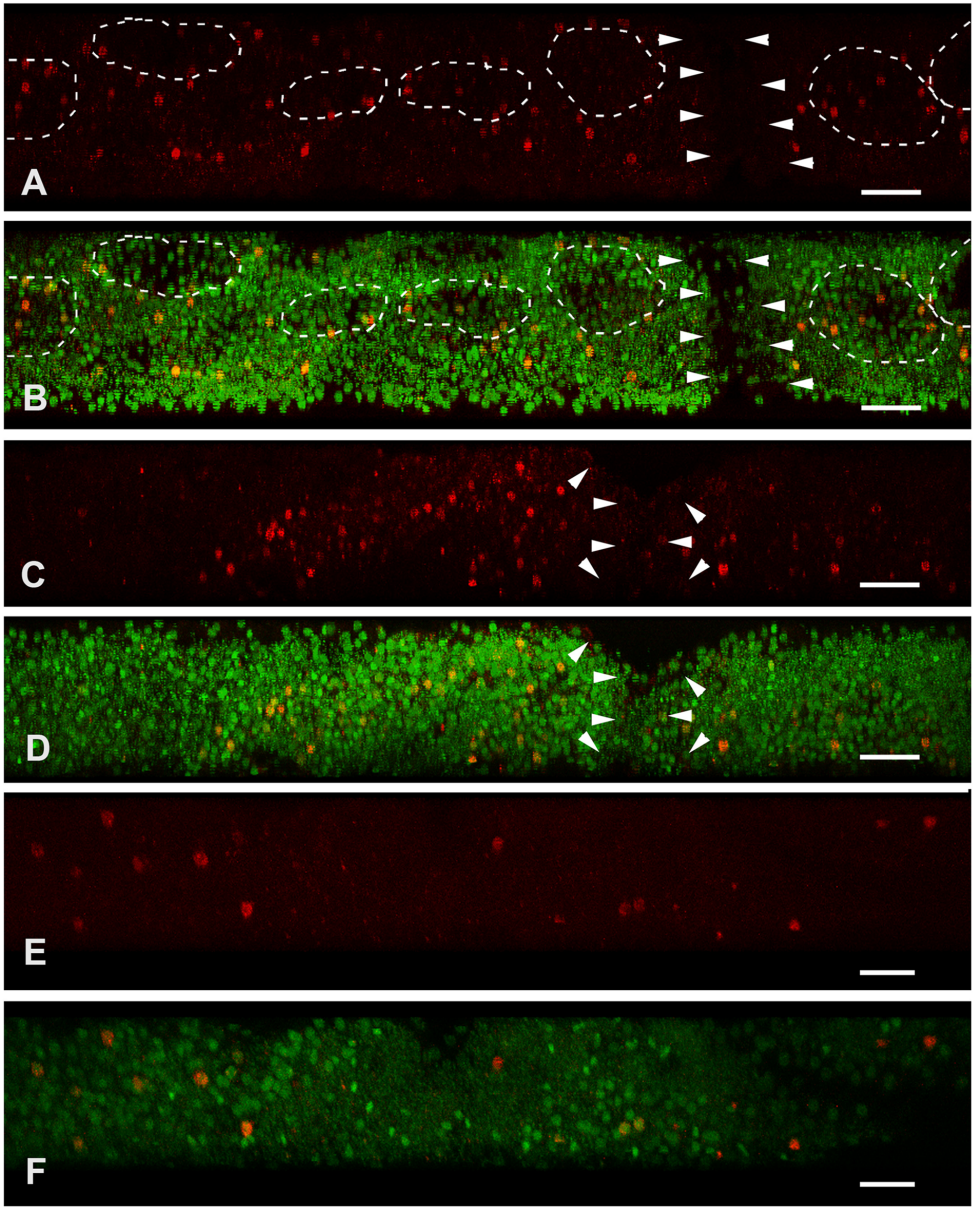
DNA synthesis in unwounded *Oscarella lobularis* (A, B) and during regeneration (C, D—stage II—12h; E, F—stage III—48h); stacks of confocal sections (3D, view at artificial transverse section). Wound surface or exopinacoderm is at the top. EdU is red, DNA is green. Arrowheads indicate aquiferous canal, choanocyte chambers highlighted by dotted line. Scale bars—20 μm, thickness of the Z-stacks 70 μm.

With the help of lateral Z-stacks of confocal images captured from sponges at different stages of regeneration, we can see that DNA-synthesizing cells distribute fairly uniformly through the sponge body (Fig 15). The small decrease of DNA synthesis observed in 48 hour regenerating samples is possibly the result of continuous incubation in a restricted volume of sea water with EdU solution.

## Discussion

### Regenerative strategies in *Oscarella lobularis*

Extensive studies of regeneration phenomena in different animals bring to light the misunderstanding of certain principles of regeneration, and first of all the classical categorization of regeneration as either “epimorphosis” or “morphallaxis”. For example, the regeneration processes vary not only between closely related taxa, but also within a single organism. A good example is the planarians blastema formation results from a mixed morphallactic-epimorphic process that varies according to the site of the amputation [29]. Other example is chordate *Amphioxus*, which oral cirri regeneration passes through morphallaxis [30], while amputated tails regenerates through an epimorphic process, with active proliferation in an blastema [31]. Based on such examples, some authors proposed doing away with this rigid division of regeneration as “epimorphosis” versus “morphallaxis” [29,32]. Following these arguments, we also do not use these therms to describe the results of our research.

In demosponges, the most investigated poriferans, regeneration relies on processes, which involves the proliferation and migration of preexisting stem cells (archaeocytes) to the injured areas (reviewed in [3,19]). In contrast to demosponges, we show that in homoscleromophs, neither blastema formation nor local dedifferentiation and proliferation are obligatory steps in the regeneration process, which is probably due to the high morphogenetic plasticity of the tissue. However, during this period (at least within the first three days after injury), the remaining exopinacoderm, endopinacoderm and choanoderm undergo transdifferentiation and differentiation.

Our study of proliferation in *Oscarella* shows that in intact sponges, choanocytes are an actively dividing cell population while pinacocytes are a static cell population which can be said to have stalled in S-phase although very slow division rate or presence of just little proliferative active fraction is also possible. In the course of the regeneration of the wound surface as well as in intact sponges, exopinacocytes is rarely labeled with EdU cells. During regeneration, the number of DNA-synthesizing cells and their distribution in the tissue in general, is not markedly changed. Thus, our data suggests that in *Oscarella*, as in *Hydra* [33], the main process for the regeneration of complete structure is not cell division, but rather, cell transdifferentiation. *Oscarella* regeneration can be qualified as the processes in which lost body parts are replaced by the remodeling of the remaining tissue, similar to *Hydra* regeneration and to the last phase of regeneration in triploblastic animals [34,35].

This mode of regeneration with the elements of metaplasia (transdifferentiation) has been described in the calcarean sponges *Sycon lingua*, *Leucosolenia complicata* [36,37], and *S*. *ciliatum* [38]. As has been seen in *Oscarella lobularis*, three main sources of new exopinacoderm have been evidenced in *S*. *lingua* and *S*. *ciliatum*: first, the neighboring exopinacoderm, second, the neighboring endopinacoderm, and third, the choanocytes of cut choanocyte chambers which transdifferentiated into new exopinacoderm with no destruction of their epithelial structure [37,38].

### Stem cells

Stem cells are characterized by their ability to renew themselves through mitotic cell division and to differentiate into a diverse range of specialized cell types [39,40].

Based mostly on histological studies, it is generally thought that the archaeocytes are likely to be the pluripotent stem cells in sponges, and that all other types of cells differentiate from archaeocytes [19,22,23,41–43]. Nevertheless, Diaz [44] proposed that the choanocytes in the demosponge *Suberites massa* have the capacity to dedifferentiate into archaeocytes. In the demosponge *Ephydatia fluviatilis*, the *EfPiwiA* gene is a gene pertaining to the Piwi family whose involvement in stem cell maintenance in eumetazoans is expressed both in archaeocytes and choanocytes. Funayama [22] thus hypothesized that in demosponges, the stem cell system is composed of two types of potentially pluripotent cells: archaeocytes and choanocytes.

The results of regeneration and somatic embryogenesis in another sponge lineage, the Calcarea, shows that the principal pluripotent cells are the choanocytes rather than the archaeocytes [3,36,37,45–47].

*Oscarella lobularis*, like some other *Oscarella* species, does not possess archaeocytes in its mesohyl [25,27,48]. It is interesting to note that in *Oscarella malakhovi*, a species that possesses archaeocytes [49], these cells are thought to be stem cells for the germ cell line [50]. We show in the present work that during formation of new exopinacoderm and upper parts of inhalant canal (endopinacoderm), the main role is played by pinacocytes and, to a lesser extent, by choanocytes. Using the Indian Ink labeling assay for detecting choanocyte line, we demonstrated that a large proportion of *O*. *lobularis* choanocytes keep the particles of Indian ink during two days without their transfer to other cells (see Additional file).

### Epithelial morphogenesis during *Oscarella* regeneration

In this work we have shown for the first time the cellular processes of regeneration for a homoscleromorph sponge. Investigations of regeneration in *Oscarella lobularis* have revealed many modes of epithelial morphogenesis in which the integrity of the epithelium is maintained (conversely to mesenchymal morphogenesis). Interestingly, these modes are similar to those observed during morphogenesis in normal development of both homoscleromorph sponges and eumetazoans.

#### Inflexion of the epithelial layers

Epithelial folding is a basic morphogenetic process of development in Eumetazoa [51]. The first step of the regenerative process in *Oscarella* is the recovering of the wound area by a dense extra cellular matrix (ECM). This observation reveals that ECM also contributes significantly to movement of the epithelial layers during *O*. *lobularis* regeneration, because interactions between epithelial cells and the ECM play a fundamental role in epithelial morphogenesis [52].

As we show here, ostia and subsequent inhalant canals are formed by epithelial invagination of the exopinacoderm during ectosomal regeneration. Whereas many cells are involved in invagination (and evagination) processes during the embryo folding and the larva metamorphosis in *Oscarella* [53–55], invagination during ostia formation involves only some of them, namely the rosettes consisting of a few exopinacocytes on the regenerate surface. This has been seen both during exopinacoderm regeneration and during sponge growth [56]. During this invagination, some of the epithelial cells involved in the bending process undergo a characteristic cell shape change: their apical ends decrease in surface area and their basal surfaces may expand, thereby producing wedge-shaped cells. The function of such wedge-shaped cells, or bottle cells, is critical for proper initiation of invagination in eumetazoans, for example, in the antozoan *Nematostella vectensis* [57], in the sea-urchin *Lytechinus pictus* ([58] or in *Xenopus laevis* ([59].

#### Changes in the area of the epithelial layer resulting from changing cell shape

Spreading and fusion of epithelial sheets involving flattening, elongation, and shortening of cells during ontogenesis in Homoscleromorpha as well as in Eumetazoa have already been widely described [55]. Clear examples of flattening in Homoscleromorpha are found during the metamorphosis of larvae and in bud formation during asexual reproduction [26,53,54]. Here we show that flattening plays an important role during reparative regeneration of exopinacoderm in *Oscarella* from choanocytes (Fig 9).

A good example of spreading and fusion in intact Homoscleromorpha can be found in egg follicle formation during the egg vitellogenesis by the endopinacocytes which surround mature eggs [60]. During this process, leading edge cells of the endopinacoderm arise at the border between two subpopulations of endopinacocytes from adjoining exhalant canals. When the epithelial sheets join, nascent junctions form by rapid contacts.

**Fig 16:**
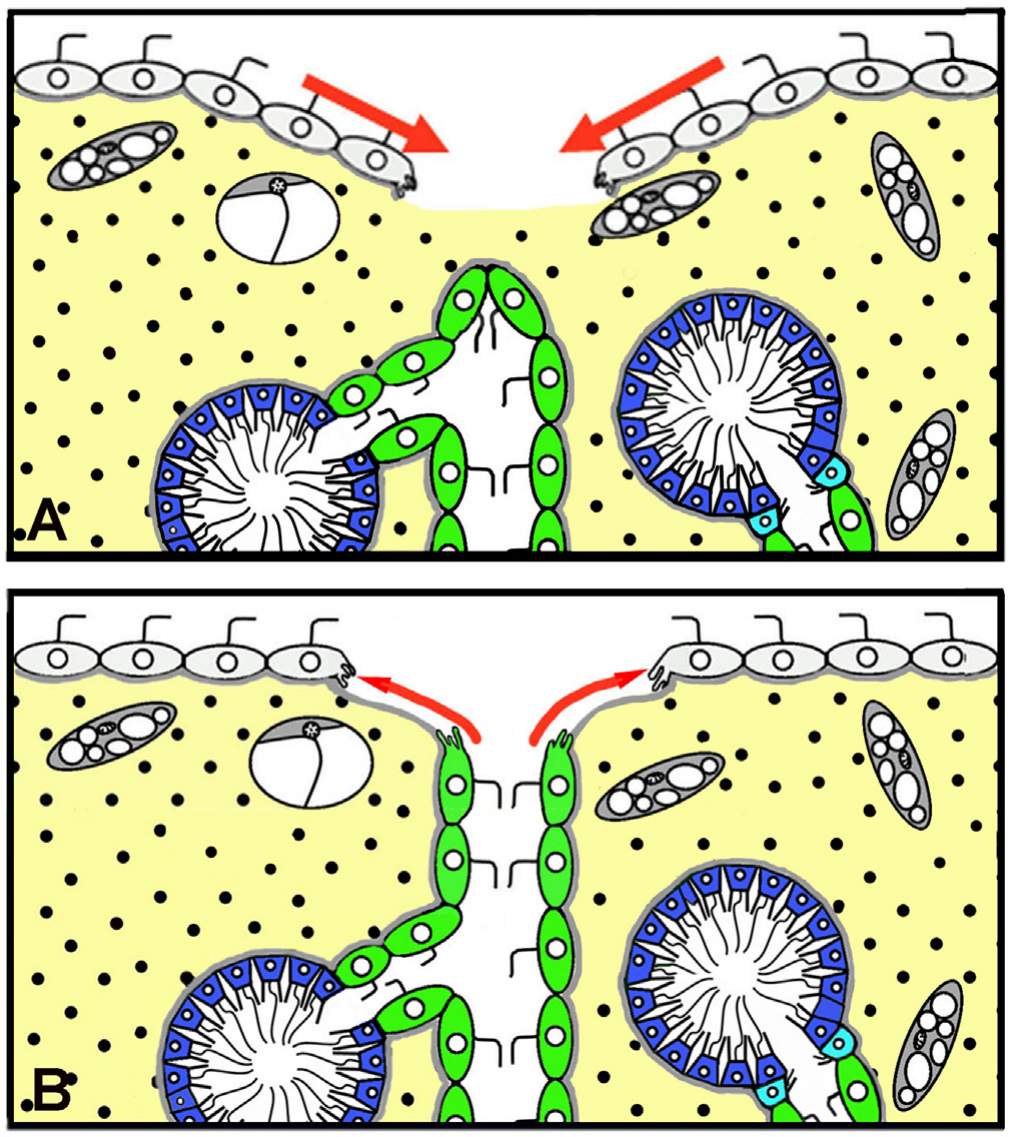
**A**. Diagram of the spreading of the layer of intact exopinacocytes, surrounding a wound during reparative regeneration of the ectosome in *Oscarella lobularis* to form new exopinacoderm. **B**. Diagram of the spreading of the layers of intact endopinacocytes from adjacent aquiferous system canals to form new exopinacoderm.

In the present work we have described the major role played by spreading and fusion in new exopinacoderm formation during reparative regeneration of the ectosome in *Oscarella*. The main source of new exopinacoderm is the layer of intact exopinacocytes surrounding a wound. The spreading of these exopinacocytes is also accompanied by their flattening. This mode of spreading entails the sliding of the exopinacocyte layer through the marginal zone to regenerate (Fig 16a) and involves a layer of endopinacocytes from adjacent aquiferous system canals (Fig 16b). In both cases, intact pinacocytes maintain the intercellular junctions and the basement membrane.

Spreading and fusion have been also described in light microscopy investigations during exopinacoderm regeneration in the demosponge *Halichondria panicea* [61,62], in the calcareous sponge *Sycon lingua* [37], and during oscular tube formation in *Ephydatia fluviatilis* [63].

As was previously shown, most morphogenesis processes in Homoscleromorpha (egg follicle development and wrinkle formation in blastula; rhagon development during metamorphosis; sponge growth starting as formation of projections of the exopinacoderm; budding; ostia formation) follow the epithelial type [25,55]. Here we show that this is also the case for reparative regeneration. This mode of epithelial morphogenesis is likely related to the true epithelial structure of homoscleromorphs.

In contrast, in other sponge lineages with cellular organization (Calcarea and Demospongiae), where cell junctions are lost [24], epithelial morphogenesis is rare during normal ontogenesis: only four examples in Calcarea and Demospongiae have been described so far: (1) incurvation of stomoblastula in calcaronean embryogenesis [64–66]; (2) invagination in prelarva of the demosponge *Halisarca dujardini* [67,68]; (3) and (4) exopinacoderm formation from the posterior pole cells in *Halisarca* disphaerula and calcaronean amphiblastula larvae metamorphosis [69,70]. However, some elements of epithelial morphogenesis have been induced owing to reparative regeneration in calcareans (*Sycon lingua* [37], *S*. *ciliatum* [38]) and in demosponges (*Halichondria panicea* [61,62], *Ephydatya fluviatilis* [63]).

## Conclusions

Regeneration is an important regulatory phenomenon with far-reaching biological implications. Regeneration involves the recognition of tissue loss or injury, followed by mechanisms that reconstruct or restore the relevant structure [71]. This phenomenon as a central discipline in biology holds great promise not only as regards the understanding of species-specific developmental issues, but also for deduction of evolutionary roots and for medical applications [9,12,71].

Although regeneration is a widespread phenomenon, it shows a heterogeneous distribution in different phyla independent of their organization and complexity level [4,12]. The evolutionary questions concerning regeneration are of primary importance in the final analysis, because they alone encompass a completely satisfying account of the phenomenon. In spite of the wide choice of potential models for studying regeneration, this phenomenon has been explored in detail in only a few animals, such as hydrozoans and planarians among invertebrates and amphibian urodeles among vertebrates [9].

Because sponges (Porifera) are widely accepted as one of the oldest metazoan lineages [15], the study of their regeneration mechanisms is expected to provide decisive clues as to the evolutionary origin of fundamental processes acting in animal development. However, until now, all investigations of sponge regeneration have been provided with traditional experimental methods under light microscopy. In spite of tremendous progress in molecular biology in recent years, we still know very little about the molecular aspects of determination and differentiation of specific cell types and the way these processes are regulated during regeneration in sponges. This is why it is necessary to investigate sponge regenerative processes with molecular tools supported by functional data and expression studies.

Knowledge of the morphological basis of morphogenesis during *Oscarella* regeneration may have important implications for our understanding of the diversity and evolution of regeneration mechanisms in metazoans and provides a strong basis for future investigations with molecular-biological approaches.

Thus, the accumulation of cellular and molecular data involved in different morphogenesis of homoscleromorphs, including regeneration, and their comparison with both other sponge lineages and with Eumetazoa, should provide important information regarding not only the origin and early evolution of regeneration mechanisms in animals, but also concerning the fundamental stem cell system and its evolution in metazoans.

## Supporting Information

S1 Fig The TEM images of the cells of *Oscarella lobularis* after 12 cultivation in Indian ink. **A**. Choanocyte with Indian ink inclusions in the phagosomes (arrows). Insert: phagosomes of a choanocyte with Indian ink particles (arrows). **B**. Exopinacocyte. **C**. Endopinacocyte. **D**. Vacuolar cell type 1. **E**. Vacuolar cell type 2. b—symbiotic bacteria, bm—basal membrane, f—flagellum, n—nucleus, ph—phagosome, v—vacuole. Scale bars: A—E—2 μm, Insert—1 μm.
(TIF)
