# Supplementary figures and images for: Oscarella lobularis (Homoscleromorpha, Porifera) Regeneration: Epithelial Morphogenesis and Metaplasia
Source: PLoS One. 2015 Aug 13;10(8):e0134566. doi: 10.1371/journal.pone.0134566 (PMC4536211; doi:10.1371/journal.pone.0134566)

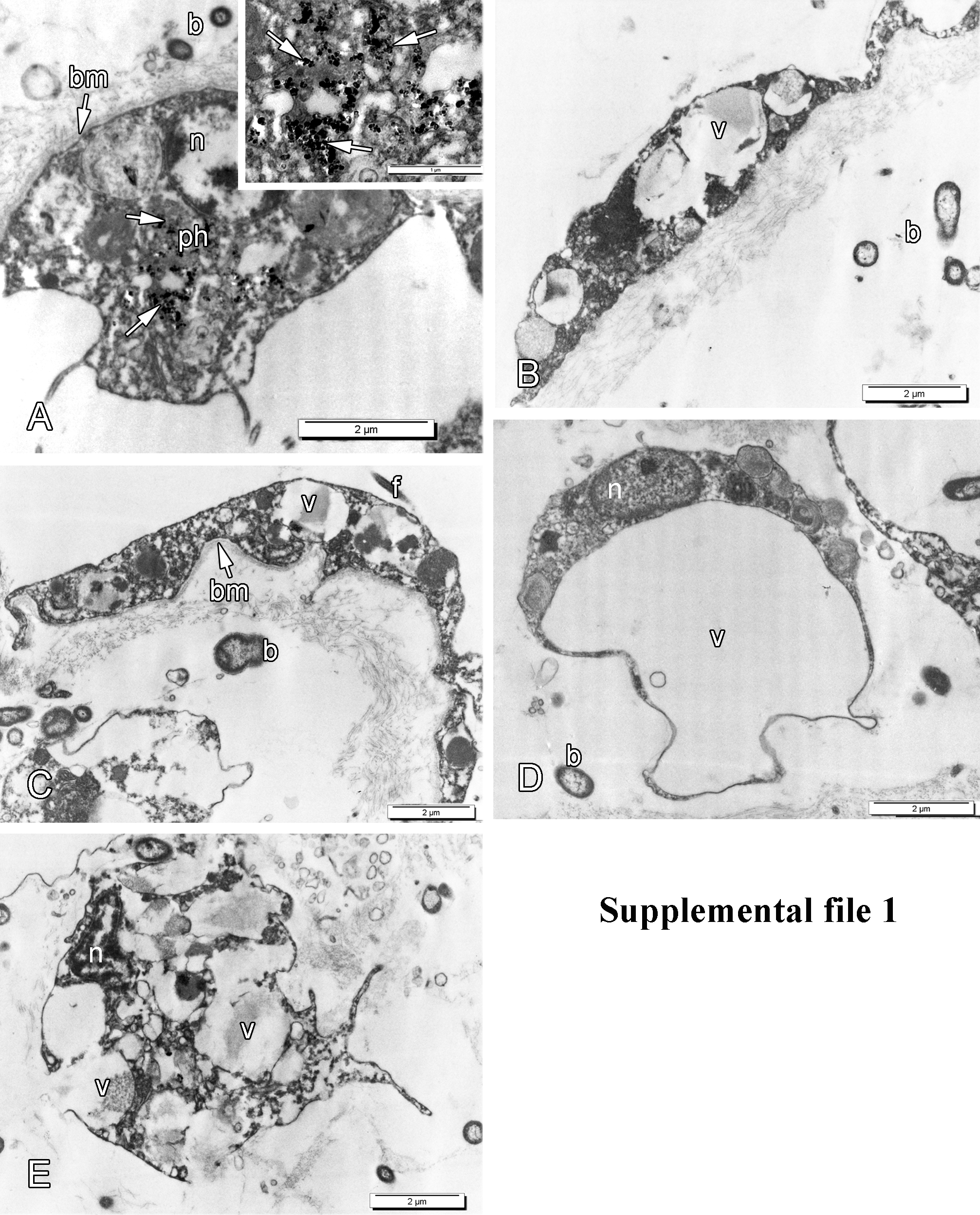

Supplement: S1 Fig — A. Choanocyte with Indian ink inclusions in the phagosomes (arrows). Insert: phagosomes of a choanocyte with Indian ink particles (arrows). B. Exopinacocyte. C. Endopinacocyte. D. Vacuolar cell type 1. E. Vacuolar cell type 2. b—symbiotic bacteria, bm—basal membrane, f—flagellum, n—nucleus, ph—phagosome, v—vacuole. Scale bars: A—E—2 μm, Insert—1 μm. (TIF) [file pone.0134566.s001.tif]
